# Aβ Assemblies Promote Amyloidogenic Processing of APP and Intracellular Accumulation of Aβ42 Through Go/Gβγ Signaling

**DOI:** 10.3389/fcell.2022.852738

**Published:** 2022-04-04

**Authors:** Magdalena Antonino, Paula Marmo, Carlos Leandro Freites, Gonzalo Emiliano Quassollo, Maria Florencia Sánchez, Alfredo Lorenzo, Elena Anahi Bignante

**Affiliations:** ^1^ Instituto de Investigación Médica Mercedes y Martín Ferreyra, INIMEC-CONICET-Universidad Nacional de Córdoba, Córdoba, Argentina; ^2^ Centro de Microscopía y Nanoscopía, CEMINCO-CONICET-Universidad Nacional de Córdoba, Córdoba, Argentina; ^3^ Institute of Biochemistry Biocenter, Goethe University Frankfurt, Frankfurt, Germany; ^4^ Instituto Universitario de Ciencias Biomédicas de Córdoba (IUCBC), Córdoba, Argentina

**Keywords:** Alzheimer’s disease, amyloid beta, amyloid precursor protein, BACE1, Gβγ subunit, gallein, human neurons, iPSc

## Abstract

Alzheimer’s disease (AD) is characterized by the deposition of aggregated species of amyloid beta (Aβ) in the brain, which leads to progressive cognitive deficits and dementia. Aβ is generated by the successive cleavage of the amyloid precursor protein (APP), first by β-site APP cleaving enzyme 1 (BACE1) and subsequently by the γ-secretase complex. Those conditions which enhace or reduce its clearance predispose to Aβ aggregation and the development of AD. *In vitro* studies have demonstrated that Aβ assemblies spark a feed-forward loop heightening Aβ production. However, the underlying mechanism remains unknown. Here, we show that oligomers and fibrils of Aβ enhance colocalization and physical interaction of APP and BACE1 in recycling endosomes of human neurons derived from induced pluripotent stem cells and other cell types, which leads to exacerbated amyloidogenic processing of APP and intracellular accumulation of Aβ42. In cells that are overexpressing the mutant forms of APP which are unable to bind Aβ or to activate Go protein, we have found that treatment with aggregated Aβ fails to increase colocalization of APP with BACE1 indicating that Aβ-APP/Go signaling is involved in this process. Moreover, inhibition of Gβγ subunit signaling with βARKct or gallein prevents Aβ-dependent interaction of APP and BACE1 in endosomes, β-processing of APP, and intracellular accumulation of Aβ42. Collectively, our findings uncover a signaling mechanism leading to a feed-forward loop of amyloidogenesis that might contribute to Aβ pathology in the early stages of AD and suggest that gallein could have therapeutic potential.

## Introduction

Cerebral deposition of amyloid beta (Aβ) is the pathologic hallmark of Alzheimer’s disease (AD), the most prevalent neurodegenerative pathology in older people (Selkoe and Hardy, 2016). Aβ is a 40–42 amino acid peptide, with a natural propensity to aggregate giving rise to pathogenic species, including oligomers and fibrils. An imbalance favoring Aβ production over its clearance leads to the accumulation of toxic Aβ assemblies in the brain and the development of AD (Selkoe and Hardy, 2016). Therefore, identifying molecular mechanisms that enhance Aβ production is a key for unveiling the pathophysiology of AD and adequate targets for rational therapies.

Aβ production begins by shedding the large extracellular portion of amyloid precursor protein (APP) by β-site APP cleaving enzyme 1 (BACE1), the rate-limiting enzyme for Aβ biosynthesis (Vassar et al., 1999). This cleavage generates a membrane-tethered APP C-terminal fragment (β-CFT) and releases a large APP N-terminal soluble fragment (sAPP-β). The intramembrane cleavage of β-CFT by γ-secretase generates Aβ, liberating the remaining APP intracellular domain (AICD) in the cytosol (Thinakaran and Koo, 2008). Thus, the necessary first step for amyloidogenesis is the convergence of APP and BACE1 in subcellular compartments with the appropriate milieu for BACE1 activity.

In different cell types, amyloidogenic processing of APP might occur in diverse subcellular compartments, including the Golgi/secretory pathway and endosomes (Rajendran et al., 2006; Sannerud et al., 2011; Haass et al., 2012). Previous studies revealed that, in neurons, BACE1 processing of APP occurs preferentially in recycling endosomes (RE) in the endocytic pathway (Das et al., 2013; Das et al., 2016). However, the signaling process and mechanism that regulate APP and BACE1 convergence and its consequent β-processing remain poorly understood. Interestingly, application of exogenous Aβ1–42 leads to intracellular accumulation of newly synthesized Aβ in the insoluble fraction of cell lysates (Yang et al., 1999). In addition, the exogenous application of Aβ assemblies to human leptomeningeal smooth muscle cells or rat neurons in culture enhances amyloidogenic processing of APP and Aβ secretion into the media (Davis-Salinas et al., 1995; Marsden et al., 2011). In line with these findings, the local application of pathologic Aβ assemblies can accelerate the spreading of Aβ deposition in the brains of APP transgenic mice (Langer et al., 2011; Walker and Jucker, 2015). These observations suggest that extracellular Aβ assemblies can activate cell surface-signaling mechanisms leading to a feed-forward process, thereby enhancing Aβ production and amyloid spreading. Consequently, elucidating the signaling mechanisms underlying this feed-forward process is crucial for understanding the progression of Aβ pathology, particularly during the early stages of AD.

Heterotrimeric guanine nucleotide-binding proteins (G proteins) are evolutionarily conserved membrane-associated proteins widely used as signal-transduction systems for a myriad of cellular events (Marinissen and Gutkind, 2001). G proteins are composed of Gα and Gβγ subunits that directly interact with cell surface G protein-coupled receptors(GPCRs) through the Gα subunit. Upon binding to its ligand, the GPCR activates G protein by exchanging GDP for GTP on Gα subunits, which causes dissociation of Gα from the GPCR and Gβγ subunits. Afterward, both Gα-GTP and Gβγ subunits can independently activate downstream signaling cascades (Marinissen and Gutkind, 2001). Conventional GPCRs are seven transmembrane domain proteins, while unconventional GPCRs are single pass membrane proteins (Marinissen and Gutkind, 2001). APP is an unconventional GPCR that can activate Go heterotrimeric protein (Nishimoto et al., 1993; Bignante et al., 2013; Copenhaver and Kogel, 2017). Significantly, toxic Aβ assemblies require APP to cause neuronal dysfunctions (Kirouac et al., 2017; Puzzo et al., 2017; Wang et al., 2017). Two different portions of the APP ectodomain can interact with Aβ species, the N-terminal portion and the Aβ-juxtamembrane domain (Van Nostrand et al., 2002; Shaked et al., 2006; Kedikian et al., 2010; Libeu et al., 2011), both of which can trigger Go activation upon binding to Aβ (Sola Vigo et al., 2009; Fogel et al., 2014). Surprisingly, no studies have analyzed the implication of Aβ–APP interaction and Go protein signaling for the amyloidogenic processing of APP.

In the present work, we analyzed the effect of Aβ assemblies on the convergence and interaction of APP and BACE1 in subcellular compartments of different cell types including human neurons derived from induced pluripotent stem cells (iPSCs), by using fluorescent biosensors and advanced microscopy techniques. We assessed the role of Aβ-APP-Go signaling pathway in the amyloidogenic processing of APP by analyzing the effects of mutant variants of APP, which are inefficient at binding Aβ or to activating Go protein. Finally, for dissecting the role of Go protein Gβγ subunits in the feed-forward mechanism of amyloidogenesis triggered by Aβ, we expressed βARKct, a universal sequester of Gβγ subunits or used gallein, a specific pharmacological inhibitor of Gβγ (Lehmann et al., 2008; Seneviratne et al., 2011). Our findings describe a signaling pathway leading to a feed-forward amyloidogenic process with high therapeutic relevance for AD.

## Material and Methods

### List of Antibodies and Reagents

Antibodies used were as follows: rabbit anti-APP C-terminal clone Y188 (1:250; Abcam, Cambridge, United Kingdom), mouse anti-neuron-specific class III β tubulin clone #TuJ1 (1:2000; Abcam, Cambridge, United Kingdom), mouse anti-MAP2 clone AP-20 (1:500; Merck Millipore; Burlington, MA, Unites States), rabbit anti-Rab11 (1:50; Thermo Fisher, Waltham, MA, United States), mouse anti-GM130 clone 35/GM130 (1:500; BD Bioscience, Franklin Lakes, NJ, United States), mouse anti-Aβ17-24 clone 4G8 (1:1,000; BioLegend, San Diego, CA, United States), mouse anti-Aβ oligomeric clones NU1 and NU4 (1:1,000; a generous gift from Dr Charles G. Glabe, University of California, Irvine, CA, United States), mouse anti-Gαo clone A2 (1:500; Santa Cruz Biotechnology, Dallas, TX, United States), mouse anti-GFP clone 3E6 (1:1,000; Molecular Probes, Eugene, OR, United States), mouse α-tubulin clone B-5-1-2 (1:1,000; Sigma-Aldrich, San Luis, MO, United States), and mouse anti-Aβ42 clone 12F4 (1:50; BioLegend, San Diego, CA, Unites States). Immunofluorescence primary antibodies were labeled with Alexa-conjugated secondary antibodies Alexa 488, 546, 598 or 633 (1:500; Invitrogen, Carlsbad, CA, US), and for western blotting primary antibodies were conjugated with secondary antibodies IR800CW and IR680RD (1:5,000, LI-COR, Lincoln, Nebraska, US) and analyzed in IR Oddissey System.

The following reagents were used: polyethyleneimine 87 K (PEI, produced by Juan M Lázaro-Martinez, Universidad Nacional de Buenos Aires, Argentina), Lipofectamine 2000 (Thermo Fisher, Waltham, MA, United States) was used according to manufacturer’s instructions, gallein (Santa Cruz Biotechnology, Dallas, TX, United States) and DAPT (Santa Cruz Biotechnology, Dallas, TX, United States) were dissolved in DMSO and applied directly to the cultures at the indicated concentrations.

### Preparation of Aβ Assemblies

Synthetic Aβ1-42, Aβ1-40, and Aβ25-35 were obtained from Biopeptide Inc. (San Diego, CA, United States). Lyophilized Aβ1-40 and Aβ1-42 peptides were dissolved in 1,1,1,3,3,3-hexafluoro-2-propanol (HFIP, Sigma-Aldrich, San Luis, MO, United States) to 1 mM and incubated at room temperature (RT) for 30 min. Thereafter, peptide solutions were aliquoted, and HFIP was evaporated at RT to allow film formation. Peptide films were stored at −20°C until use. To prepare fibrillar Aβ1-40 or Aβ1-42, peptide films were resuspended in sterile double-distilled water to a concentration of 1 mM and incubated at 37°C for 72 h. Thereafter, peptide solution was further diluted to 500 µM in 2x phosphate-buffered saline (PBS), incubated at 37°C for 24 h and was used for treatments. To prepare fibrillar Aβ25-35, lyophilized peptide was dissolved in sterile double-distilled water to a concentration of 1 mM and further diluted in PBS to 500 µM and incubated for 1 day at 37°C. Oligomeric Aβ1-42 was prepared as described by Stine et al. (2003). Briefly, Aβ1-42 films were resuspended in dimethyl sulfoxide (DMSO, Sigma-Aldrich, San Luis, MO, United States) to 5 mM, vortexed, and sonicated for 10 min. Afterward, cold phenol-free F-12 culture media was added to a final concentration of 100 μM Aβ and incubated for 24 h at 4°C. Thereafter, Aβ solution was centrifuged at 4°C for 10 min at 14,000 × g, and the supernatant was used for treatments. Monomeric Aβ was prepared by resuspending the Aβ1-42 film in sterile water reaching a final concentration of 100 μM Aβ. The conformation of Aβ monomers and oligomers was confirmed by dot blot analysis, with antibodies NU1 and NU4 that specifically recognize Aβ oligomers and antibody 4G8 that recognizes Aβ independently of its conformation.

### DNA Constructs

The plasmid used for cell transfection were as follows: pcDNA3.1 (Life Technologies) (empty vector e.v.) as control or encoding full-length wild-type human APP695 (APP) generated in our laboratory, encoding β-adrenergic receptor kinase C-terminal peptide (βARKct) provided by Alfredo Cáceres, Instituto de Investigación Médica Mercedes y Martín Ferreyra, INIMEC-CONICET-UNC, Córdoba, Argentina, encoding wild-type human BACE1 (BACE1) provided by Wim Annaert, Catholic University of Leuven, Leuven, Belgium, encoding BACE1 fused to cherry fluorescent protein (BACE1:CH), APP:VN and BACE1:VC provided by Subhojit Roy, University of California, San Diego, La Jolla, CA, United States, encoding Rab11 fuse to red fluorescent protein (Rab11:RFP) provided by Cecilia Conde, Instituto de Investigación Médica Mercedes y Martín Ferreyra, INIMEC-CONICET-UNC, Córdoba, Argentina. Full-length human APP695 wild-type and mutant forms (APP:YFP, APP∆β:YFP, and APPGP:YFP) were inserted into pEYFP-N3 encoding the yellow fluorescent protein (YFP, Clontech, Mountain View, CA, United States). APP∆β was generated by deletion of the juxtamembrane domain (aa 597-624) of human APP695 and was previously characterized (Sola Vigo et al., 2009). APPGP contains a double substitution of H657 and H658 by G and P, respectively. The mutations were introduced into human APP695 by using the QuikChange Site-Directed Mutagenesis Kit (Stratagene, San Diego, CA, United States) and confirmed by sequencing. This double-mutation prevents APP-dependent activation of Go protein (Nishimoto et al., 1993; Brouillet et al., 1999).

### Culture of Cell Lines

Mice neuroblastona cells N2a and human epithelial cervical carcinoma HeLa cells (American Type Culture Collection (ATCC)) were cultured in DMEM supplemented with 10% fetal bovine serum (FBS) (Gibco, Thermo Fisher, Waltham, MA, United States) and GlutaMAX (Gibco, Thermo Fisher, Waltham, MA, United States). Cell cultures were maintained at 37°C in a 5% CO_2_ humidified atmosphere.

### Culture of Human-Induced Pluripotent Stem Cells

hiPSc were cultured on a feeder layer of irradiated mouse embryonic fibroblasts (iMEFs) in iPSc media DMEM/F12 (Gibco, Thermo Fisher, Waltham, MA, United States) supplemented with NEAA (Gibco, Thermo Fisher, Waltham, MA, United States), GlutaMAX (Gibco, Thermo Fisher, Waltham, MA, United States), 10% KSR (Gibco, Thermo Fisher, Waltham, MA, United States), 100 µM 2-mercaptoethanol (0.25 mg/ml, Sigma-Aldrich), 10 mM HEPES (0.25 mg/ml, Sigma-Aldrich), 20 ng/ml bFGF (PeproTech, Cranbury, NJ. United States), and Pen/Strep (Gibco, Thermo Fisher, Waltham, MA, United States) and maintained at 37°C in a 5% CO_2_ atmosphere. Media was replaced every 2 days, and the cells were split every 5–6 days based on colony growth. Differentiating colonies were removed from the plate prior to splitting. For passaging, cells were dissociated with collagenase.

### Neuronal Differentiation From hiPSc

For the induction of neurons from hiPSc, we followed an embryoid body-based protocol described by Zeng et al. (2010). Briefly, hiPSc colonies were dissociated and cultured as aggregates in suspension in iPSc media with 10 µM Y27632 (Tocris, Bristol, United Kingdom) and 4 ng/ml bFGF (PeproTech, Cranbury, NJ. United States). After 3 days, aggregates were switched to neural induction media (NIM) (DMEM/F12 (Gibco, Thermo Fisher, Waltham, MA, US), supplemented with NEAA (Gibco, Thermo Fisher, Waltham, MA, United States), GlutaMAX (Gibco, Thermo Fisher, Waltham, MA, United States), N2 (Gibco, Thermo Fisher, Waltham, MA, United States), 1 mg/ml heparin (Sigma-Aldrich, San Luis, MO, United States), 4 ng/ml bFGF, and Pen/Strep (Gibco, Thermo Fisher, Waltham, MA, United States). After 7–10 days, cell aggregates were plated on Geltrex-coated dishes (ThermoFisher, Waltham, MA, United States) in NIM media with 20 ng/ml bFGF (Peprotech, Cranbury, NJ, United States). Primitive neuroepithelial (NE) structures form over 7–10 days and more than 17 days neural rosettes were present. Neural rosettes were selected manually for further expansion on Geltrex-coated dishes and grown in neural differentiation media (NDM). Neurobasal supplemented with NEAA, GlutaMAX, N2, B27, 1 µM cAMP, 10 ng/ml BDNF (PeproTech, Cranbury, NJ, United States), 10 ng/ml GDNF (PeproTech, Cranbury, NJ, United States), 10 ng/ml IGF-1 (PeproTech, Cranbury, NJ, United States), 200 ng/ml ascorbic acid, and Pen–Strep were used in the study. Media was replaced every other day. For final neuronal differentiation, dissociated rossettes were cultured in Geltrex-coated coverslips in Neurobasal supplemented with NEAA, GlutaMAX, N2, B27, and Pen–Strep and were maintained in this media until experimental use. This protocol yields a culture mainly composed of human neurons and astrocytes (Supplementary Figure S3).

### Cell Transfection

N2a and HeLa cells grown at 70–80% confluence in 35 mm dishes were transfected by directly adding a mixture of 0.5 μg of indicated DNA and 0.05 mM PEI dissolved in NaCl 0.15 mM to the culture media, and after 24–48 h, the cultures were analyzed.

Transfection of human neurons for APP/BACE1, bimolecular fluorescent complementation (BiFC) assay was carried out in 10 DIV cultures of human neurons derived from hiPSCs growing in 35 mm dishes. The conditioned media was replaced by a transfection mixture composed of 4 µg plasmid encoding APP:VN, 4 µg plasmid encoding BACE1:VC, and 10 µl of Lipofectamine 2000 (Thermo Fisher) in 1.0 ml of Opti-MEM (Gibco, Thermo Fisher, Waltham, MA, United States). After 2 h, the transfection mixture was removed, the original-conditioned media was restituted, and cultures were analyzed after 48 h.

### Immunofluorescence

Immunolabeling was performed as described previously (Bignante et al., 2018). Briefly, cultures grown in coverslips were fixed with 4% paraformaldehyde, 0.12 M sucrose in PBS for 20 min at 37°C, permeabilized for 5 min with 0.2% Triton X-100 in PBS, blocked for 1 h in 5% horse serum (HS), and incubated overnight at 4°C with primary antibody. Afterward, the coverslips were incubated with Alexa-conjugated secondary antibodies for 1 h at room temperature and with 2.5 µM DAPI for 5 min for nuclei stain. Finally, coverslips were mounted on slides with Fluorsave Reagent (Calbiochem). For intracellular Aβ1-42 immunodetection, cells were permeabilized with 0.1% saponin for 1 h and blocked in PBS containing 10% HS at room temperature for 1.5 h. Then, incubation with primary (clone 12F4) and secondary anti-mouse Alexa 488 antibodies, DAPI staining and mounting were performed using the standard procedure described earlier (adapted from Almeida et al., 2006; Almeida et al., 2006).

### Analysis of Fluorescence and Colocalization Quantitation

All fluorescent images, including those for colocalization and/or BiFC experiments, were captured in either, an Olympus FV1000 spectral or an LSM 800 Zeiss confocal microscope, equipped with a PLAPON ×60 oil objective. Z-stack images were acquired with the optical cuts of 0.12 µm. The images were deconvolved and projected on the *Z*-axis for intensity measurement. Fluorescent intensity was determined by using Fiji software (NIH). For colocalization analysis, images of each pair of proteins to be analyzed were open in separate channels and submitted to a background subtraction. An ROI was traced in channel one, and Manders correlation index M1 (Bolte and Cordelieres, 2006) was obtained by running the plugin “Coloc 2” from Fiji software. Thereafter, Manders coefficients were used to calculate the percentage of APP, APP∆β, APPGP, and BACE1 in different organelles and BiFC in RE. Identical settings for image capture and analysis were conserved across all samples of the same experiment. For human neurons derived from iPSc, images were obtained from the soma and neurites with a 4× optical zoom, which were identified by MAP2 or βIII-tubulin staining.

### Assessment of Cell Viability of N2a Cells and Human iPSC-Derived Neurons

The cell viability of N2a and human neurons was assessed by counting viable and pyknotic nuclei after staining with fluorescent DAPI. Pyknotic nuclei, characteristic of apoptotic cells, presented a smaller, condensed, and more brilliant aspect with respect to viable nuclei. Images were obtained at ×40 in a confocal fluorescent microscope LSM 800 (F-Fluar oil ×40 objective). Three random fields were analyzed per coverslip, and nine coverslips were scored per condition from three different experiments. Values were expressed as percentage of viable cells (total minus apoptotic cells) respect to total cells. To avoid bias during quantitative analysis, all samples were coded, and determinations were performed under a treatment-blind condition.

### Analysis of Protein Expression by Western Blot

Cultures of N2a cells were transfected with empty vector (control) or APP:VN and BACE1:VC constructs, and after 1 h, the cultures were treated with the γ-secretase inhibitor DAPT (10 µM), gallein (5 µM), o-Aβ (1 µM), or the corresponding vehicles. After 48 h, cultures were lysed in RIPA buffer supplemented with a protease inhibitor cocktail (SIGMAFAST, Sigma) at 4°C. Cell lysates were diluted in Laemmli sample buffer, incubated at 95°C for 5 min, resolved in 12% gel by SDS-PAGE, and electrotransferred onto a nitrocellulose membrane. Thereafter, the membrane was boiled for 5 min in PBS and incubated with agitation overnight at 4°C with anti-APP C-terminal antibody (clone Y188) or anti-α-tubulin (clone B-5-1-2) followed by the corresponding IR800CW/IR680DR secondary antibodies. Thereafter, membranes were scanned and visualized using the Odyssey Systems. Quantitative densitometric analysis of the bands was performed using Fiji software. CTFs levels were normalized against α-tubulin.

### Characterization of Aβ Assemblies by Dot Blot

To characterize the conformation of Aβ preparations, we used dot blot analysis with antibodies NU1 and NU4 that specifically recognize oligomeric conformations of Aβ42 or antibody 4G8 that recognizes Aβ independently of its conformation. Aβ monomers of 1 µg (m-Aβ), Aβ fibrils (f-Aβ), or Aβ oligomers (o-Aβ) were seeded onto a nitrocellulose membrane. After 5 min, the membrane was blocked with 5% horse serum in PBS for 1 h and incubated overnight at 4°C shaking with 4G8, NU1, or NU4 antibodies. Afterward, membranes were incubated with the IR800CW secondary antibody for 1 h at room temperature and visualized by the IR Odyssey Systems.

### Statistical Analysis

Each experiment was independently replicated two−four times. Experimental data were statistically analyzed using *t*-test or ANOVA followed by LSD Fisher *post-hoc* test, and *p* ≤ 0.05 was considered as statistically significant. For non-parametric data, Mann–Whitney U test or Kruskal–Wallis test was run followed by Dunn *post-hoc* test. The results are presented as mean ± SEM.

## Results

### Aβ Enhances APP and BACE1 Convergence in Recycling Endosomes by a Signaling Pathway Mediated by Go Protein Gβγ Subunit in Cell Lines

**FIGURE 1:**
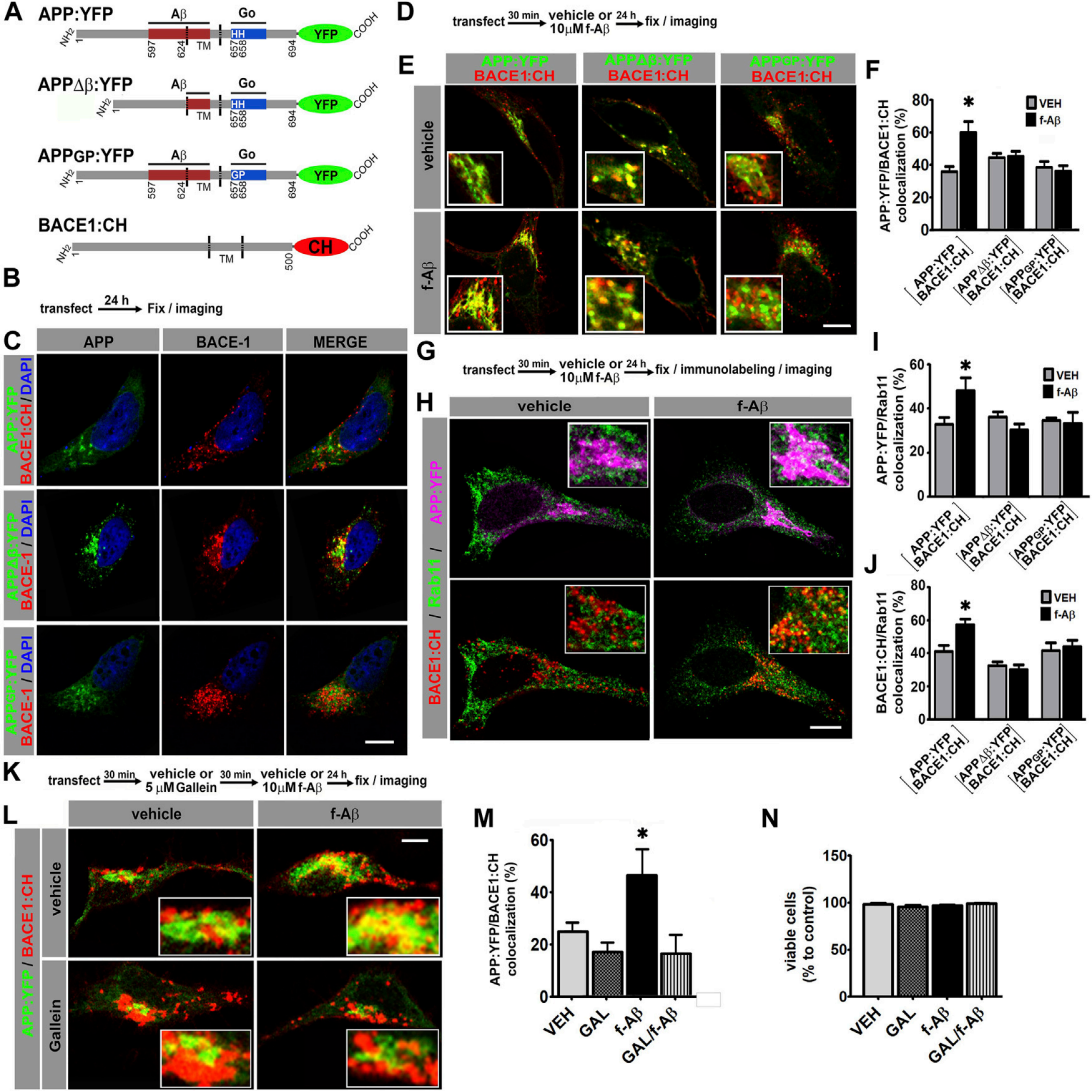
Fibrillar Aβ increases colocalization of APP:YFP and BACE1:CH in recycling endosomes through Go/Gβγ-dependent signaling in HeLa and N2a cells. **(A)**. Schematic illustration of variants of APP695 fused to YFP and BACE1 fused to CH. Aβ and Go protein-interacting domains are indicated as red and blue boxes, respectively. Amino acid numbering for APP and BACE1 are indicated. TM, transmembrane domain. **(B)**. Experimental diagram for C. **(C)**. Confocal images of HeLa cells co-expressing the indicated proteins. **(D)**. Experimental diagram for E and F. **(E)**. Confocal images of HeLa cells co-expressing the indicated proteins. **(F)**. Manders coefficients of colocalization of APP:YFP, APP∆β:YFP, or APPGP:YFP and BACE1:CH expressed as percent (n = 56 between 8 and 11 cells for each condition) *: *p* ≤ 0.001 vs. VEH by ANOVA followed by LSD *post-hoc* test. **(G)**. Experimental diagram for H-J. **(H)**. Confocal images of HeLa cells co-transfected with the indicated proteins and immunolabeled with antibody against Rab11 followed by Alexa Fluor 633-conjugated antirabbit antibody. Upper panel shows pseudocolor images for APP:YFP (magenta) and Rab11(green), and lower panel for BACE1:CH (red) and Rab11 (green). **(I)**. Manders coefficients of colocalization of APP:YFP and Rab11 expressed as percent (n = 62 between 8 and11 cells for each condition) *: *p* ≤ 0.005 vs. VEH by ANOVA followed by LSD *post-hoc* test. **(J)**. Manders coefficients of colocalization of BACE1:CH and Rab11 expressed as percent (n = 62; between 8 and11 cells for each condition) *p = 0.001 vs. VEH by ANOVA followed by LSD *post-hoc* test. **(K)**. Experimental diagram for L-N. **(L)**. Confocal images of N2a cells co-expressing the indicated proteins. **(M)**. Manders coefficients of colocalization of APP:YFP and BACE1:CH expressed as percent (n = 29; between 7 and 8 cells for each condition) *: *p* = 0.004 vs. GAL + f-Aβ and *p* = 0.031 vs. VEH by ANOVA followed by LSD *post-hoc* test. **(N)**. Quantitative analysis of viable cells expressed as percent of control (n = 27, between 6 and 7 cells for each condition). In all cases, data represent mean ± SEM. Scale bar is 10 μm. VEH: vehicle (control); f-Aβ: fibrillar Aβ1-42; Gal: gallein. Inserts are ×2.5 enlargement of the corresponding image.

We have previously characterized in detail that binding of Aβ fibrils (f-Aβ) to cell surface APP specifically activates Go protein (Kedikian et al., 2010; Sola Vigo et al., 2009). To examine the role of APP–Go protein signaling in amyloidogenic processing, we analyzed subcellular localization of fluorescently tagged wild-type or mutant forms of APP and BACE1 in culture cells by confocal laser scanning microscopy and performed quantitative colocalization analysis. Cherry fluorescent protein was fused to human BACE1 (BACE1:CH), while yellow fluorescent protein was fused to wild-type or mutant human APP (APP:YFP) (Figure 1A). One APP mutant version lacks the extracellular Aβ domain (APPΔβ:YFP), which prevents the binding of APP to f-Aβ (Shaked et al., 2006; Sola Vigo et al., 2009). The other APP mutant version contains the substitution of histidines at positions 657 and 658 by glycine and proline (APP_GP_:YFP), respectively, which impairs APP’s ability to activate Go protein signaling (Nishimoto et al., 1993; Brouillet et al., 1999).

HeLa cells are commonly used for cell biology studies due to their big size and flat morphology, which facilitate protein colocalization analysis. In addition, HeLa cells have been extensively used for studying APP processing. We co-transfected HeLa cells with BACE1:CH and APP:YFP, APPΔβ:YFP, or APP_GP_:YFP and evaluated the subcellular localization of APP and BACE1 by confocal microscopy (Figures 1B,C). Consistent with previous reports, wild-type and mutant forms of APP:YFP showed a cytoplasmic expression with a high condensation in the perinuclear area in a compartment whose structure is similar compared to the Golgi apparatus, while BACE1:CH depicted a punctate expression throughout the cytoplasm (Sannerud et al., 2011; Haass et al., 2012; Chia et al., 2013; Jiang et al., 2014) (Figure 1C; Supplementary Figure S1A).

In the AD brain, f-Aβ accumulates at high levels within amyloid plaques, which are loci of degenerating tissue. To evaluate how this pathological condition might alter APP and BACE1 colocalization, we transfected HeLa cells with BACE1:CH and wild-type or mutant forms of APP:YFP and treated the cultures with vehicle (control) or 10 µM f-Aβ, to model the environmental milieu in the proximities of amyloid plaques. After 24 h, cultures were fixed, and colocalization of APP:YFP and BACE1:CH was analyzed (Figures 1D,E). We found that f-Aβ significantly increased colocalization of BACE1:CH and APP:YFP, but not between BACE1:CH and APPΔβ:YFP or APP_GP_:YFP (F (5,51) = 4.77, *p* = 0.001; Figures 1E,F). Therefore, in HeLa cells f-Aβ enhances colocalization of wild-type APP and BACE1, an effect that requires the Aβ juxtamembrane sequence of APP and the histidine doublet required for activating Go protein signaling.

Although interaction of APP with BACE1 and amyloidogenic processing may occur in different subcellular compartments, we focused on RE and the Golgi/secretory pathway. Neuronal activity induces β-processing of wild-type APP preferentially in RE (Das et al., 2013), while pathogenic Swedish APP mutation (APPSWE) dramatically enhances amyloidogenic processing in the Golgi apparatus/secretory pathway (Haass et al., 1995; Thinakaran et al., 1996). To test whether f-Aβ enhances APP and BACE1 colocalization in RE and/or in the Golgi-secretory pathway, we co-transfected HeLa cells with BACE1:CH and wild-type or mutant APP:YFP. Afterward, we treated the cultures with vehicle or 10 µM f-Aβ, and 24 h later, we fixed the cultures and performed immunolabeling for Rab11 or GM130, which are resident proteins of the RE and Golgi apparatus, respectively (Ullrich et al., 1996; Nakamura et al., 1995). In control conditions we observed similar colocalization of wild-type and mutant forms of APP:YFP with Rab11 (Figures 1G–I and Supplementary Figures S1B–D). Colocalization of APP:YFP or APPΔβ:YFP with GM130 was also similar under control conditions (Supplementary Figures S1E,F). These observations confirmed that these APP mutations did not grossly affect the subcellular distribution of the protein in non-polarized HeLa cells. On the other hand, BACE1:CH depicted a punctate expression throughout the cytoplasm, which is consistent with its enrichment in the endosomal compartment (Chia et al., 2013). In control condition, BACE1:CH distribution was similar regardless of whether it was co-expressed with wild-type or mutant variants of APP (Figures 1C,H,J; Supplementary Figures S1A–D). Interestingly, we observed that f-Aβ treatment significantly enhanced simultaneous colocalization of APP:YFP with Rab11 (F (5,57) = 2.907, *p* = 0.021; Figures 1H,I) and BACE1:CH with Rab11 (F (5; 57) = 7.795; *p* = 0.001; Figures 1H,J). We also found that f-Aβ treatment enhanced colocalization of APP:YFP with GM130 (F (3,37) = 9.303; *p* = 0.001; Supplementary Figures S1E,F). However, it was not accompanied by an incremented colocalization of BACE1:CH in this compartment (F (3,37) = 1,800; *p* = 0.164; Supplementary Figures S1E,G). Notably, in cells co-transfected with BACE1:CH and APPΔβ:YFP or APPGP:YFP, we observed that f-Aβ treatment did not alter colocalization of either APP or BACE1 with Rab11 (Figures 1I,J and Supplementary Figures S1C,D). Moreover, in cells transfected with APPΔβ:YFP and BACE1:CH colocalization with GM130 was not altered either (Supplementary Figures S1E,G). Thus, in HeLa cells f-Aβ selectively enhances colocalization of APP and BACE1 in Rab11-positive RE, which requires an APP-juxtamembrane Aβ domain for binding to f-Aβ and the integrity of the APP sequence for activating Go protein signaling.

We have recently found that Gβγ subunits signaling, rather than Gαo subunit, mediates APP-dependent toxicity of f-Aβ in primary neurons (Bignante et al., 2018). Thus, to determine in a neuronal cell line whether f-Aβ enhances colocalization of APP and BACE1 by activating an APP-dependent signaling through Go protein and Gβγ subunits, we used gallein, a small molecule that specifically binds to Gβγ subunits preventing its interaction with downstream signaling effectors (Lehmann et al., 2008; Seneviratne et al., 2011). We co-transfected neuroblastoma N2a cells with APP:YFP and BACE1:CH. We pre-incubated the cultures with vehicle (control) or 5 µM gallein for 30 min and subsequently with vehicle or 10 µM f-Aβ. After 24 h we fixed the cultures and assessed APP and BACE1 colocalization by confocal microscopy. We found that gallein completely abrogated the enhanced colocalization of APP:YFP and BACE1:CH in cultures treated with f-Aβ (F (3,26) = 4.479; *p* = 0.012; Figures 1K–M). To evaluate whether altered distribution of APP and BACE1 might be associated with enhanced cell death due to f-Aβ or gallein treatments, we analyzed cell viability by scoring the proportion of normal vs. fragmented and/or condensed nuclei stained with DAPI, which are well-established characteristics of apoptotic cells. We found that the viability of N2a cells treated with 10 µM f-Aβ and/or 5 µM gallein was not significantly different from vehicle-treated cultures (F (1,24) = 1.882; *p* = 0.183, Figure 1N). Altogether, these experiments indicate that f-Aβ enhances colocalization of APP and BACE1 in RE of HeLa and N2a cells. In these neuronal cell lines, this effect was associated with Go protein Gβγ subunits signaling and not with cell death.

### Pathologic Aβ Assemblies Increase Physical Interaction of APP and BACE1 Through Gβγ Signaling

**FIGURE 2:**
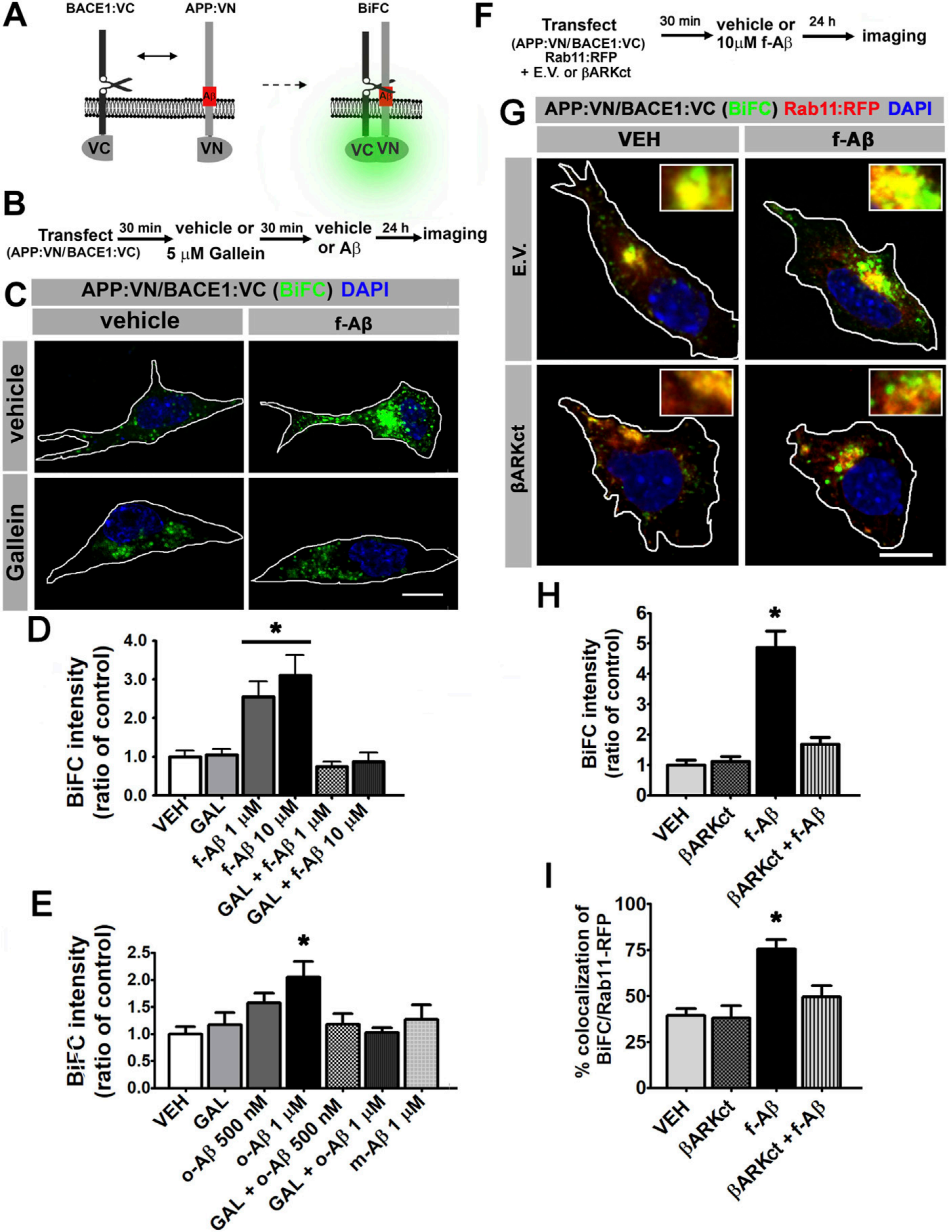
Fibrillar and oligomeric Aβ enhance the physical interaction of APP and BACE1 through Gβγ-dependent signaling in N2a cells. **(A)**. Scheme of bimolecular fluorescent complementation (BiFC) system for detecting APP-BACE1 interaction. Interaction of APP and BACE1 fused to complementary fragments (VN and VC) of Venus fluorescent protein reconstitutes fluorescence. **(B)**. Experimental diagram for C-E. **(C)**. Representative fluorescent images of BiFC in N2a cultures co-transfected with plasmid encoding to APP:VN and BACE1:VC and treated under the indicated conditions. **(D)**. BiFC intensity expressed as ratio of control (n = 65; 8–19 cells for each condition) *: *p* < 0.001 vs. all other conditions by ANOVA followed by Fisher LSD *post-hoc* test. **(E)**. BiFC intensity expressed as ratio of control (n = 108; 12–18 cells for each condition) *: *p* < 0.01 vs. VEH and vs. o-Aβ 1uM + GAL by ANOVA followed by Fisher LSD *post-hoc* test. **(F)**. Experimental diagram for G-I. **(G)**. Representative confocal fluorescent images of BiFC and Rab11:RFP in N2A cells co-transfected with βARK or empty vector; and treated with the indicated conditions. **(H)**. BiFC intensity expressed as ratio of control (n = 46; 10–13 cells for each condition) *: *p* < 0.05 vs. all other conditions by Kruskal–Wallis followed by Dunn *post-hoc* test. **(I)**. Manders coefficients of colocalization of BiFC and Rab11:RFP, expressed as percent (n = 44; 10–13 cells for each condition). *: *p* < 0.01 vs. all other conditions by ANOVA followed Fisher LSD *post-hoc* test. In all cases data represent mean ± SEM. Scale bar is 10 μm.

Amyloidogenic processing requires the physical interaction of APP with BACE1. To determine whether APP and BACE1 colocalization induced by f-Aβ is effectively associated with increased physical interaction between the proteins, we used fluorescence bimolecular complementation (BiFC). BiFC is a validated technique for detecting interaction between a protein pair, in which one protein of the pair is fused to the N-terminal fragment of the Venus fluorescent protein (VN), and the other protein is fused to the complementary C-terminus (VC). VN or VC is not fluorescent; however, when the protein pair physically interacts, Venus is reconstituted and becomes fluorescent (Kerppola, 2006). An additional advantage of this technique is that complementation of Venus is irreversible and therefore transient protein–protein interactions are stabilized, allowing its visualization. Here, we used expression vectors for BiFC which are designed and characterized for studying APP and BACE1 interactions in living neurons (Das et al., 2016). Briefly, one vector drives the expression of wild-type APP tagged with VN (APP:VN), while the other induces the expression of BACE1 tagged with VC (BACE1:VC) (Figure 2A). To determine the effect of f-Aβ and the role of Gβγ subunits signaling on the physical interaction between APP and BACE1, we co-transfected N2a cells with APP:VN and BACE1:VC vectors. After incubation with vehicle (control) or 5 µM gallein for 30 min, we added vehicle, 1 or 10 µM f-Aβ and fixed the cultures 48 h later (Figure 2B). In control cultures, we noticed that BiFC became evident as fluorescent dots throughout the cytoplasm. Treatment with both doses of f-Aβ significantly enhanced BiFC fluorescence, while gallein completely normalized BiFC intensity to control levels (Figure 2C). Quantitative assessment confirmed a significant enhancement of APP–BACE1 BiFC intensity in f-Aβ-treated cultures and its blockage by gallein (F (2,46) = 5.058; *p* = 0.001; Figure 2D).

Conversion of physiologic non-aggregated Aβ to pathologic Aβ assemblies, including Aβ oligomers and fibrils, plays a key role in neuronal dysfunction in AD. Therefore, it is important to determine whether the enhanced convergence of APP and BACE1 induced by f-Aβ might also be promoted by other Aβ species. We generated non-aggregated soluble Aβ (m-Aβ) and aggregated soluble oligomers (o-Aβ) (Stine et al., 2003) and characterized these Aβ species by dot blot with 4G8, NU1, and NU4 antibodies (Lambert et al., 2007). This analysis confirmed that our o-Aβ preparation contains misfolded species that are recognized by the conformational-dependent antibodies, NU1 and NU4 (Supplementary Figure S2). Then, we transfected N2a cells with APP:VN and BACE1:VC and treated them with vehicle or 5 µM gallein, and 30 min later we applied vehicle or 500 nM or 1 µM o-Aβ or 1 μM m-Aβ. After 24 h we fixed the cultures and analyzed BiFC intensity. We found that o-Aβ, but not m-Aβ, induced a dose-dependent increase in BiFC that reached significance at 1 µM. Moreover, gallein efficiently abrogated the enhancement in BiFC induced by o-Aβ (F (3,106) = 3.663; *p* = 0.016; Figure 2E). Thus, pathologic assemblies of Aβ, including oligomers and fibrils, enhance the physical interaction of APP and BACE1 by a mechanism dependent on Gβγ signaling.

Next, we employed βARKct to confirm that the increase in APP and BACE1 interaction is triggered by Gβγ subunits. βARKct contains the Gβγ-binding domain of β-adrenergic receptor kinase 1 and, when expressed in intact cells, it inhibits Gβγ-dependent signaling by binding to and sequestering Gβγ subunits (Koch et al., 1994). We performed multiple transfections of N2a cells with plasmids encoding for APP:VN, BACE1:VC, and Rab11 fused to the red fluorescent protein (Rab11:RFP) together with the vector encoding βARKct or the corresponding empty vector (E.V.). Afterward, we applied vehicle (control) or 10 µM f-Aβ, and 24 h later we fixed the cultures and performed analysis of BiFC and Rab11-RFP fluorescence (Figures 2F,G). In agreement with our previous results, we found that BiFC was significantly enhanced by f-Aβ. We also noticed that the expression of βARKct completely abrogated this effect, an observation that was confirmed by quantitative analysis (H (3) = 31,472; *p* = 0.001; Figure 2H). Moreover, we performed colocalization analysis of BiFC and Rab11-RFP and found that it was significantly enhanced by treatment with f-Aβ and abolished by the expression of βARKct (F (3,41) = 12.537, *p* = 0.001; Figure 2I). These experiments strongly support the conclusion that pathologic assemblies of Aβ enhance the physical interaction of APP and BACE1 in RE by a Gβγ subunit-dependent signaling mechanism.

### Pathologic Aβ Assemblies Trigger Amyloidogenic Processing of APP and Intracellular Accumulation of Aβ42 by a Gβγ-Signaling-Dependent Mechanism

**FIGURE 3:**
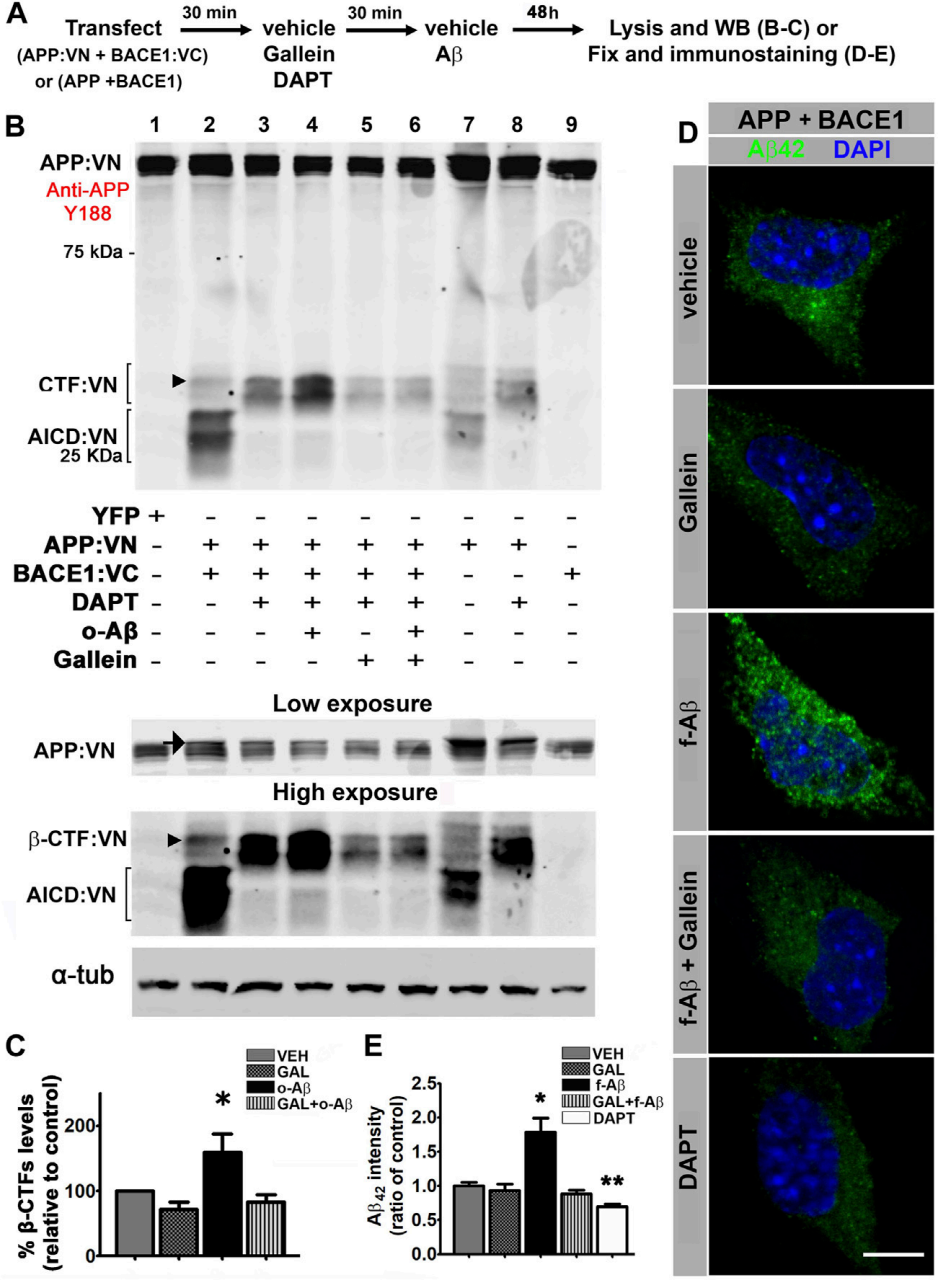
Fibrillar and oligomeric Aβ enhance amyloidogenic processing of APP and promote intracellular accumulation of Aβ42 through a Gβγ-dependent signaling in N2a cells. **(A)**. Experimental diagram for B-E. **(B)**. Cell lysates of N2a cells co-transfected with APP:VN and BACE1:VC and treated under the indicated conditions were analyzed by Western blot with antibody Y188 that recognize the C-terminal domain of APP. Bands corresponding to CTF:VN and AICD:VN are indicated by brackets. Molecular weight markers are indicated in kDa. Low and high exposures of the same blot are shown below to allow a better visualization of bands corresponding to APP: VN (low exposure) and β-CTF:VN (high exposure), which enabled their better identification for quantification. Arrow in the low-exposure panel points to full-length APP:VN. Arrowheads point to β-CTF:VN. Loading control α-tubulin is shown in the bottom. **(C)**. Quantitative densitometry analysis of β-CTF:VN bands after normalization of their levels to those of corresponding loading control band (n = 4) *: *p* < 0.05 vs. all other conditions by ANOVA followed Fisher LSD *post-hoc* test. In all cases values denote mean ± SEM. **(D)**. Confocal images of N2a cells co-transfected with wild-type APP and BACE1 plasmids, inmunolabeled with antibody to Aβ42 and treated under the indicated conditions. **(E)**. Aβ42 fluorescence intensity expressed as ratio of control (n = 55; 10–12 cells for each condition) *: *p* ≤ 0.05. In all cases, data represent mean ± SEM. Scale bar is 10 μm.

To find out whether APP-BACE1 interaction genuinely correlates with β-processing of APP, we expressed APP:VN and BACE1:VC and analyzed a BACE1-cleaved C-terminal fragment of APP (β-CTF) by Western blot. If BACE1:VC effectively cleaves the APP:VN protein, a β-CTF linked to the 17–19 kDa VN protein (β-CTF:VN) should be generated. In addition, cleavage of β-CTF:VN by γ-secretase should generate APP:VN intracellular domain fragments (AICD:VN). Hence, if γ-secretase activity is blocked by the inhibitor DAPT (10 µM), β-CTF:VN should accumulate. We transfected N2a cultures with APP:VN and BACE1:VC and treated them with corresponding vehicles, 10 µM DAPT, 5 µM gallein, and/or 1 µM o-Aβ. After 48 h we harvested the cells and analyzed the lysates by Western blot with an antibody against the APP C-terminal domain (Figure 3A). We observed that under control condition, expression of APP:VN alone produced two groups of bands (Figure 3B, lane 7). The lower group was abolished by DAPT (Figure 3B, lane 8), indicating that it corresponds to AICD:VN. On the contrary, the upper group was enhanced by DAPT treatment indicating that it corresponds to CTF:VN. In these experimental conditions, β-processing is due to endogenous BACE1. Co-expression of BACE1:VC with APP:VN (Figure 3B, lane 2), increased the intensity of both groups of bands, consistent with an enhanced β-processing of APP:VN by BACE1:VC. Again, DAPT completely abrogated γ-secretase processing, preventing the generation of AICD:VN and leading to enhanced accumulation of CTF:VN (Figure 3B, lane 3). These bands are undoubtedly BACE1:VC-dependent and therefore correspond to β-CTF:VN (Figure 3B, arrowhead). Importantly, treatment with o-Aβ dramatically increased β-CTF:VN (Figure 3B, lane 4) which was abolished by gallein (Figure 3B, lane 6) (F (1,14) = 5.501, *p* = 0.015; Figure 3C). Gallein *per se* also appears to reduce the basal generation of CTF:VN, but this effect lacks statistical significance (Figure 3B, lane 5 vs. lane 3). These findings demonstrate that pathological assemblies of Aβ enhance amyloidogenic processing of APP, which requires Gβγ subunits signaling.

To confirm that enhanced β-processing of APP by pathological Aβ assemblies effectively leads to an increase in Aβ generation by a mechanism that requires Gβγ subunits signaling. We transfected N2a cells with vectors encoding for human APP and BACE1 and applied corresponding vehicle (control) and/or 5 µM gallein and/or 10 µM f-Aβ (Figure 3A). After 48 h of treatment, we fixed the cultures and used antibody 12F4 to specifically immunolabel Aβ42 (Almeida et al., 2006; Burrinha et al., 2021) (Supplementary Figures S2C,D). To avoid interference of exogenously added f-Aβ with immunolabeling of endogenously generated Aβ42, in this experiment we used fibrils prepared with Aβ25-35 peptide, which is not recognized by antibody 12F4 (Supplementary Figure S2D). In control cells, we observed that antibody 12F4 depicted a faint punctate pattern throughout the cytoplasm, which was notably enhanced in cultures treated with f-Aβ25-35, while gallein reversed this effect (Figure 3D). This observation was confirmed by quantitative analysis of 12F4 fluorescence intensity (F (4,51) = 15.094, *p* = 0.001, Figure 3E). In addition, we observed a significant reduction of 12F4 fluorescence in cultures treated with 10 µM DAPT (Figures 3D,E), indicating that inhibition of γ-secretase activity precluded the generation of Aβ peptide, leading to the accumulation of CTFs which are not recognized by antibody 12F4. These experiments indicate that in N2a cells overexpressing APP and BACE1, exogenous application of f-Aβ stimulates Gβγ subunits-dependent amyloidogenic processing of APP enhancing intracellular accumulation of Aβ42 peptide.

### Pathologic Aβ Assemblies Promote an Increase in APP:VN and BACE1:VC Interaction in RE, and Intracellular Accumulation of Aβ42 in Human iPSc-Derived Neurons Through Gβγ Signaling

**FIGURE 4:**
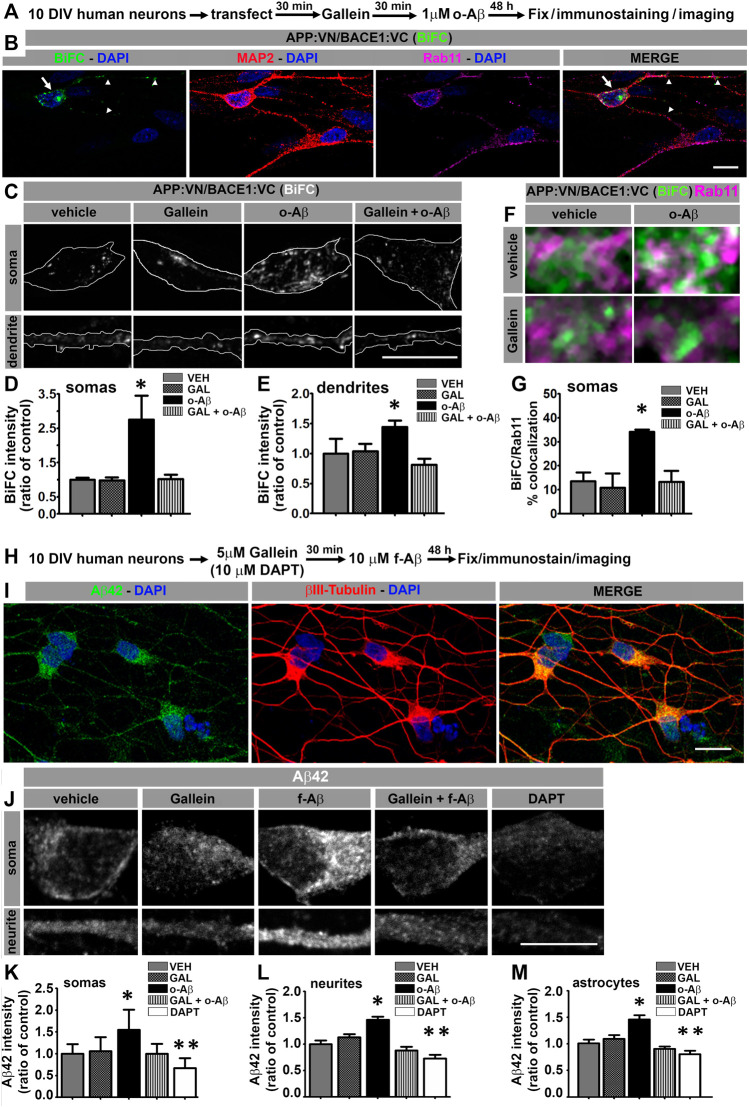
Aβ assemblies promote APP:VN/BACE1:VC interaction and intracellular Aβ42 accumulation in human iPSC-derived neurons through Gβγ-dependent signaling. **(A)**. Experimental diagram for B-G. **(B)**. Confocal image of 12 DIV human neurons immunolabeled with antibodies to MAP2 and Rab11 depicting BiFC of APP:VN and BACE1:VC. BiFC in cell body and dendrites is indicated with arrows and arrowheads, respectively. Nuclei were stained with DAPI. **(C)**. Representative images of magnified (×3) areas of somas and dendrites of human neurons treated under the indicated conditions showing BiFC of APP:VN and BACE1:VC. **(D)**. Intensity of BiFC in somas expressed as ratio of control (n = 27; 6–9 cells for each condition) *: *p* < 0.001 vs. all other conditions by ANOVA followed Fisher LSD *post-hoc* test. **(E)**. Intensity of BiFC of APP:VN and BACE1:VC in dendrites expressed as ratio of control (n = 52; 11–14 cell for each condition) *: *p* < 0.05 vs. all other conditions by ANOVA followed Fisher LSD *post-hoc* test. **(F)**. Representative images of the magnified area (×5) of somas of human neurons treated under the indicated conditions and depicting BiFC (green) and endogenous Rab11 (magenta). White pixels indicate colocalization. **(G)**. Manders coefficient of colocalization of BiFC and Rab11 expressed as percentage. Data represent means ± SEM. (n = 27; 6–9 cells for each condition). *: *p* < 0.01 by ANOVA followed by Fisher LSD *post-hoc* test. **(H)**. Experimental diagram for I-M. **(I)**. Representative confocal images of 12 DIV human neurons treated with f-Aβ25-35 10 µM and immunolabeled with antibodies to Aβ42 (clone 12F4) and βIII-tubulin. **(J)**. Representative images of the magnified area (×3) of somas and dendrites of human neurons treated with the indicated conditions showing Aβ42 immunostaining. **(K)**. Quantitative analysis of Aβ42 fluorescence intensity in somas expressed as ratio of control (n = 641, 104–175 cells for each condition) *: *p* < 0.05 vs. all other conditions by Kruskal–Wallis followed by Dunn *post-hoc* test **: *p* < 0.05 DAPT vs. VEH, GAL and f-Aβ. **(L)**. Quantitative analysis of Aβ42 fluorescence intensity in processes expressed as ratio of control (n = 155, 27–36 cells for each condition) *: *p* < 0.05 vs. all other conditions by ANOVA followed by Fisher LSD *post-hoc* test. **(M)**. Quantitative analysis of Aβ42 fluorescence intensity in astrocytes expressed as ratio of control (n = 124; 22–28 cells for each condition) *: *p* < 0.001 vs. all other conditions by ANOVA followed by Fisher LSD *post-hoc* test. **: *p* < 0.05 DAPT vs. CON, GAL and f-Aβ. In all cases, data represent mean ± SEM. Scale bar is 10 μm.

For further appraising, the pathophysiological significance of our observations and the clinical potential of modulating Gβγ signaling for AD, we analyzed the effect of o-Aβ and gallein on APP/BACE1 interaction in human neurons. Neuronal cultures derived from human iPSc were generated (Supplementary Figure S3) and transfected at 10 DIV with expression vectors for APP:VN and BACE1:VC. Next, we incubated the cultures with vehicle, 5 µM gallein and/or 1 µM o-Aβ. After 48 h of treatment, cultures were fixed and immunostained against MAP2 and Rab11 proteins (Figures 4A,B). Quantitative assessment of BiFC intensity in MAP2-positive neurons showed that o-Aβ significantly increased APP:VN and BACE1:VC interaction as evidenced by enhanced BiFC intensity in both, the cell soma (F (1,23) = 4.429; *p* = 0.046) and dendrites (F (1,48) = 5.213; *p* = 0.027), an effect that was abrogated by gallein (Figures 4B–E). Notably, o-Aβ treatment significantly increased BiFC colocalization with Rab11, effect that was abolished by gallein (F (1,23) = 4.63; *p* = 0.042; Figures 4F,G). Therefore, in human neurons o-Aβ enhances the physical interaction of APP and BACE1 in RE by a mechanism that involves Gβγ signaling.

To determine whether deposition of Aβ assemblies enhances intracellular accumulation of Aβ42 in human neurons expressing endogenous protein and to address the involvement of Gβγ subunits signaling in this process, we treated 10 DIV neuronal cultures derived from human iPSC with vehicle or 5 µM gallein and/or 10 µM f-Aβ prepared with Aβ25-35 peptide. After 48 h, we fixed and immunolabeled the cultures with 12F4 and βIII tubulin antibodies (Figures 4H,I). We performed quantitative analysis of 12F4 fluorescence and found that f-Aβ significantly enhanced fluorescence intensity in both soma (H (4) = 331.54; *p* = 0.001) and neurites (F (4,151) = 6.83; *p* = 0.001), which was abolished by gallein (Figures 4J–L). In addition, we observed that DAPT treatment significantly reduced the labeling of 12F4 antibody, which confirms the specificity of the immunolabeling (Almeida et al., 2006). It is important to note that the viability of human neurons was not significantly affected after Aβ treatments (Supplementary Figure S4) discarding the possibility that the effects described were related to neuronal death.

Finally, since our protocol for differentiating human iPSc allows the generation of neurons and astrocytes (see Supplementary Figure S3), we explored whether Aβ assemblies might also affect the amyloidogenic processing of APP in astrocytes. In control cultures, we observed that 12F4 fluorescence intensity was significantly lower in astrocytes compared to neurons (t = −3.043; *p* = 0.004), which is consistent with previous reports, indicating that neurons are the main producers of Aβ peptide (Zhao et al., 2011). In addition, we found a significant increase in 12F4 fluorescence intensity in astrocytes treated with f-Aβ and the abrogation by gallein (F (4,120) = 13.787; *p* = 0.001; Figure 4M). Collectively, these findings uncover the signaling mechanism through which pathological Aβ assemblies trigger a feed-forward process of amyloidogenesis and intracellular accumulation of Aβ42 peptide that might contribute to an imbalance between Aβ production and clearance in AD pathology.

## Discussion

This work elucidates the cellular and molecular mechanisms through which pathological Aβ assemblies stimulate the first and most critical step for Aβ production: the encounter and processing of APP by BACE1 in endosomal compartments for the generation of βCFTs, which lead to intracellular accumulation of Aβ42. We demonstrate that, in diverse cellular types including human iPSc-derived neurons, pathological Aβ assemblies drive increased APP/BACE1 interaction and amyloidogenic processing by activating an APP-dependent signaling pathway engaging Gβγ subunits of Go protein. Gallein, a specific Gβγ inhibitor, greatly attenuates the exacerbated β-processing of APP and prevents intracellular accumulation of Aβ42 triggered by Aβ assemblies. Our work provides evidence for the potential value of targeting Gβγ signaling for AD therapeutics.

A growing body of evidence indicates that physiological and pathological effects triggered by Aβ require APP(Lorenzo et al., 2000; Van Nostrand et al., 2002; Shaked et al., 2006; Sola Vigo et al., 2009; Kedikian et al., 2010; Libeu et al., 2011; Bignante et al., 2013; Fogel et al., 2014; Kirouac et al., 2017; Puzzo et al., 2017; Wang et al., 2017). In this work, we add further evidence to these observations by showing that Aβ oligomers and fibrils stimulate β-processing of APP and promote intracellular accumulation of Aβ42 through a process that requires the integrity of APP and Gβγ subunits signaling. In fact, we show that APP∆β:YFP, which lacks the juxtamembrane Aβ sequence for Aβ binding (Shaked et al., 2006; Sola Vigo et al., 2009; Kedikian et al., 2010), was ineffective in promoting convergence of APP and BACE1 in the presence of Aβ assemblies. Similarly, APPGP:YFP, an APP mutant ineffective at activating Go protein (Nishimoto et al., 1993; Brouillet et al., 1999), completely abrogated APP and BACE1 convergence induced by Aβ. The most straightforward interpretation of these data is that APP acts as an unconventional GPCR that responds to Aβ through Go protein activation. The fact that these APP mutants prevent not only their own subcellular redistribution in response to Aβ but also that of BACE1 reinforces this interpretation. Moreover, inhibition of Gβγ subunits with βARKct or gallein precluded the enhanced APP and BACE1 interaction induced by pathological forms of Aβ, adding further support to previous reports indicates that APP–Go signaling depends on Gβγ subunits (Bignante et al., 2018). Significantly, we also found that gallein prevented intracellular accumulation of Aβ42 in cultures treated with f-Aβ, including human neurons and astrocytes-expressing endogenous levels of proteins; which strengthens the physiopathological importance of our observations.

Intracellular distribution and traffic of APP and secretase have a pivotal role in amyloidogenic processing and pathogenesis of AD. It was postulated that healthy neurons maintain a fine regulation of intracellular traffic to limit APP and BACE1 proximity and Aβ production (Das et al., 2013). In neurons, APP and BACE1 colocalize in several intracellular compartments of the secretory pathway including the Golgi apparatus. However, β-cleavage of wild-type APP might not preferentially occur in the secretory pathway due to the abundance of other substrates with much higher affinity for BACE1 (Ben Halima et al., 2016). Consistent with this, studies conducted in neurons have found that BACE1 processing of APP occurs mainly in endocytic compartments and that neuronal activity promotes the convergence of APP and BACE1 in RE, thereby, increasing β-cleavage of APP (Das et al., 2013; Das et al., 2016). In line with these observations, it was reported that neuronal activity enhances Aβ secretion *in vivo* (Kamenetz et al., 2003; Cirrito et al., 2005; Brody et al., 2008), suggesting a physiological role for Aβ in synaptic transmission. In fact, physiologically secreted Aβ binds to APP modulating neurotransmitter release in excitatory synapses by a Go protein Gβγ subunits-signaling process (Fogel et al., 2014). Remarkably, enhanced encounter of APP and BACE1 in RE was also reported in neurons of the AD brain, suggesting that pathological conditions in AD favor abnormal amyloidogenic processing of APP (Das et al., 2013). Our immuno-colocalization and BiFC experiments provide a mechanistic explanation for the later observation by showing that pathological Aβ oligomers and fibrils increase the interaction of APP and BACE1 in Rab11-positive compartments by activating an APP-dependent Go protein Gβγ subunits-signaling pathway.

Our findings do not exclude the possibility that Aβ might also promote the physical interaction of APP and BACE1 in other subcellular compartments. In fact, we also found that in HeLa cells f-Aβ enhance the levels of APP:YFP, but not BACE1:CH, in the Golgi apparatus. We cannot discard that increasing the availability of APP might suffice for boosting its β-processing in the Golgi/secretory pathway. For example, β-processing of pathologic Swedish APP mutation, which has higher affinity for BACE1 than that of wild-type APP and causes early onset AD (Kinoshita et al., 2003), was reported in the Golgi apparatus and secretory pathway (Haass et al., 1995; Thinakaran et al., 1996). Thus, in addition to RE it is possible that Aβ assemblies could also favor amyloidogenic processing of APP in other subcellular compartments. Incremented colocalization of APP and BACE1 in endosomes suggests that Aβ assemblies alter their intracellular trafficking; however, other cellular processes cannot be excluded (Heredia et al., 2004; Faghihi et al., 2008; Marsden et al., 2011; Sadleir and Vassar, 2012; Mamada et al., 2015). Regardless of the cellular mechanism implicated, our experiments show that gallein effectively prevents Aβ-induced amyloidogenic processing of APP and intracellular accumulation of Aβ42. This observation indicates that Gβγ subunits signaling plays a critical role in orchestrating the pathological loop by which Aβ assemblies enhance production and intracellular accumulation of Aβ42.

Many groups have reported intracellular accumulation of Aβ in neurons of transgenic mice models of AD and in the brains of patients with mild cognitive impairment and AD (Takahashi et al., 2017). By immuno-EM intracellular Aβ42 was localized in multivesicular bodies associated with endosomal organelles (Takahashi et al., 2002), observations that are consistent with the generation of this intracellular pool of Aβ in the endocytic pathway. Moreover, intracellular Aβ was observed not only in neurons but also in astrocytes, particularly in the proximity of Aβ plaques (Kurt et al., 1999; Thal et al., 1999), suggesting a potential relation between extracellular and intracellular Aβ. In some APP overexpressing transgenic mice, a rise in intraneuronal Aβ42 levels in dystrophic neurites preceded plaque formation (Oddo et al., 2003), which led to the suggestion that plaques might originate as remnants of degenerating neurites. In this work, we provide new evidence showing that Aβ oligomers and fibrils when deposited extracellularly can trigger intracellular production and accumulation of Aβ42 in cultured human neurons-expressing endogenous protein. Hence, both intracellular and extracellular Aβ can influence each other to uphold the spreading of Aβ pathology in the AD brain. The fact that our data show that interfering with Gβγ subunits signaling can ameliorate Aβ42 accumulation in cultured cells opens a new avenue of research for testing the therapeutic potential of this signaling cascade in transgenic models of AD.

AD pathology develops with a lengthy and silent preclinical phase, in which Aβ deposition disseminates throughout the brain in a systematic and stereotyped fashion (Thal et al., 2002; Bateman et al., 2012), which presumably involves transynaptic dissemination of Aβ pathology (Lazarov et al., 2002; Harris et al., 2010). In this work, we uncover a signaling mechanism underlying a feed-forward process that exacerbates the amyloidogenic processing of APP and intracellular accumulation of Aβ42. We postulate that pathological Aβ assemblies, which are resistant to degradation, cause a sustained activation of APP–Go–Gβγ subunits signaling cascade boosting intracellular accumulation of Aβ42. Over time, this feed-forward process foster deposition and dissemination of Aβ pathology in the AD brain. Therefore, this process might be relevant for both early- and late-onset AD (EOAD and LOAD, respectively). Hence, targeting the APP/Go/Gβγ-signaling pathway may have therapeutic potential for halting Aβ pathology in both forms of the disease. Moreover, based on the evidence that targeting GPCR or their downstream-signaling cascade is the most successful pharmacological strategy for dealing with most of the human pathologies (Hauser et al., 2017); we propose that the intervention of this pathway at different levels could have therapeutic relevance. Effectiveness of gallein in modulating this process *in vitro* is the first proof of principle that targeting this signaling cascade could be a new therapeutic approach for AD treatment.

## Data Availability

The raw data supporting the conclusions of this article will be made available by the authors, without undue reservation.

## Supplementary Material

Supplementary Figure S1 Fibrillar Aβ did not alter the localization of mutant variants of APP in recycling endosomes and Golgi apparatus in HeLa cells. **(A)**. Low magnification images of N2a cells transfected with the indicated plasmids. **(B)**. Experimental diagram for C-F. Transfected HeLa cells were immunolabeled with antibodies against Rab11 or GM130 followed by Alexa Fluor 633-conjugated antibody (Invitrogen) and imaged by confocal microscopy. **(C and D)**. Images of HeLa cells co-expressing the indicated proteins and immunolabeled against Rab11. Upper panels show pseudocolor images for Rab11 (green) and APPΔβ:YFP (magenta in **C**) or APPGP:YFP (magenta in **D**). Lower panels show pseudocolor images for BACE1:CH (red) and Rab11 (green). Quantitative analysis is shown in Figure 1 **(I** and **J)**. **(E)**. Images of HeLa cells co-expressing the indicated proteins and immunolabeled against GM130. Upper panel shows pseudocolor images for APP:YFP (magenta) and GM130 (green). Lower panel shows pseudocolor images for BACE1:CH (red) and GM130 (green). **(F)**. Manders coefficient of colocalization of APP:YFP and GM130 is expressed as percentage. (n = 41; 8-12 cells for each condition) *: p < 0.001 vs. all other conditions by ANOVA followed by Fisher LSD *post-hoc* test. **(G)**. Manders coefficient of colocalization of BACE1:CH and GM130 is expressed as percentage. (n = 41; 8-12 cells for each condition) No significant difference among conditions was found by ANOVA. Inserts are x2.5 enlargement of the corresponding image. Scale bar is 10 μm.

Supplementary Figure S2 Characterization of Aβ species and 12F4 antibody specificity by dot blot. Expression of APP:VN and BACE1:VC plasmids in N2a cells.**(A)**. Dot blot analysis of Aβ species used in this work. Soluble non-aggregated Aβ1-42 (m Aβ), Aβ1-42 oligomers (o-Aβ), and Aβ1-42 fibrils (f-Aβ). Upper panel was revealed with the conformation-independent monoclonal antibody 4G8 that recognizes all Aβ species. Middle and lower panels were stained with conformation-dependent antibodies NU1 and NU4, which selectively label oligomeric species. Note that NU1 strongly labeled o-Aβ, while weakly recognized f-Aβ. NU4 antibody only labeled o-Aβ. **(B)**. Transfected N2a cells with APP:VN and BACE:VC plasmids and treated as indicated. Cultures were fixed and immunostained with antibody against GFP (Molecular Probes) followed by Alexa Fluor 598-conjugated antibody (Invitrogen) and imaged by confocal microscopy. Left panel depicts GFP stain, and right panel depicts BiFC. Note that for similar level of expression of fluorescent protein (anti-GFP stain), level of BiFC intensity varies according to treatment. Also, gallein and Aβ treatments did not affect the expression of fluorescent protein. Scale bar is 10 µm. **(C)**. Equal amounts of monomers of Aβ42 were analyzed by dot blot using 12F4 antibody at specified concentrations. **(D)**. Indicated peptides (1 µg) were analyzed by dot blot using 12F4 antibody. Note that 12F4 antibody specifically labeled Aβ42.

Supplementary Figure S3 Establishment and characterization of human neuronal cultures. **(A)**. Schematic protocol for differentiating human-induced pluripotent stem cells (iPScs) to neurons. Phase contrast images of an iPSC colony, embryonic bodies (EB) in suspension, neural epithelium (NE) and neural rosettes, and differentiated neurons are shown. Scale bar is 50 µm. **(B)**. Confocal images of human neuronal cultures at 21 DIV immunolabed for the indicated proteins. Scale bar is 10 µm.

Supplementary Figure S4 Effect of f-Aβ on viability of iPSc-derived human neurons. **(A)**. Phase contrast images of cultures of human neurons derived from iPSc treated with vehicle or 10 mM f-Aβ25-35 are shown. Upper panel 20x lower panel ×40. **(B)**. Viability was assessed by scoring viable and apoptotic nuclei stained with DAPI (ANOVA F(1,4) = 0.27607, P = 0.627). Scale bar is 50 µm.

## References

[B1] Almeida C. G. Takahashi R. H. Gouras G. K. (2006). Beta-amyloid Accumulation Impairs Multivesicular Body Sorting by Inhibiting the Ubiquitin-Proteasome System. J. Neurosci. 26, 4277–4288. 10.1523/jneurosci.5078-05.2006 16624948 PMC6673997

[B2] Bateman R. J. Xiong C. Benzinger T. L. S. Fagan A. M. Goate A. Fox N. C. (2012). Clinical and Biomarker Changes in Dominantly Inherited Alzheimer's Disease. N. Engl. J. Med. 367, 795–804. 10.1056/nejmoa1202753 22784036 PMC3474597

[B3] Ben Halima S. Mishra S. Raja K. M. P. Willem M. Baici A. Simons K. (2016). Specific Inhibition of β-Secretase Processing of the Alzheimer Disease Amyloid Precursor Protein. Cell Rep 14, 2127–2141. 10.1016/j.celrep.2016.01.076 26923602

[B4] Bignante E. A. Heredia F. Morfini G. Lorenzo A. (2013). Amyloid β Precursor Protein as a Molecular Target for Amyloid β-induced Neuronal Degeneration in Alzheimer's Disease. Neurobiol. Aging 34, 2525–2537. 10.1016/j.neurobiolaging.2013.04.021 23714735 PMC3762679

[B5] Bignante E. A. Ponce N. E. Heredia F. Musso J. Krawczyk M. C. Millán J. (2018). APP/Go Protein Gβγ-Complex Signaling Mediates Aβ Degeneration and Cognitive Impairment in Alzheimer's Disease Models. Neurobiol. Aging 64, 44–57. 10.1016/j.neurobiolaging.2017.12.013 29331876

[B6] Bolte S. Cordelières F. P. (2006). A Guided Tour into Subcellular Colocalization Analysis in Light Microscopy. J. Microsc. 224, 213–232. 10.1111/j.1365-2818.2006.01706.x 17210054

[B7] Brody D. L. Magnoni S. Schwetye K. E. Spinner M. L. Esparza T. J. Stocchetti N. (2008). Amyloid-β Dynamics Correlate with Neurological Status in the Injured Human Brain. Science 321, 1221–1224. 10.1126/science.1161591 18755980 PMC2577829

[B8] Brouillet E. Trembleau A. Galanaud D. Volovitch M. Bouillot C. Valenza C. (1999). The Amyloid Precursor Protein Interacts with GoHeterotrimeric Protein within a Cell Compartment Specialized in Signal Transduction. J. Neurosci. 19, 1717–1727. 10.1523/jneurosci.19-05-01717.1999 10024358 PMC6782156

[B9] Burrinha T. Martinsson I. Gomes R. Terrasso A. P. Gouras G. K. Almeida C. G. (2021). Upregulation of APP Endocytosis by Neuronal Aging Drives Amyloid-dependent Synapse Loss. J. Cell Sci 134, jcs255752. 10.1242/jcs.255752 33910234

[B10] Chia P. Z. C. Toh W. H. Sharples R. Gasnereau I. Hill A. F. Gleeson P. A. (2013). Intracellular Itinerary of Internalised β-Secretase, BACE1, and its Potential Impact on β-Amyloid Peptide Biogenesis. Traffic 14, 997–1013. 10.1111/tra.12088 23773724

[B11] Cirrito J. R. Yamada K. A. Finn M. B. Sloviter R. S. Bales K. R. May P. C. (2005). Synaptic Activity Regulates Interstitial Fluid Amyloid-β Levels *In Vivo* . Neuron 48, 913–922. 10.1016/j.neuron.2005.10.028 16364896

[B12] Copenhaver P. F. Kögel D. (2017). Role of APP Interactions with Heterotrimeric G Proteins: Physiological Functions and Pathological Consequences. Front. Mol. Neurosci. 10, 3. 10.3389/fnmol.2017.00003 28197070 PMC5281615

[B13] Das U. Scott D. A. Ganguly A. Koo E. H. Tang Y. Roy S. (2013). Activity-induced Convergence of APP and BACE-1 in Acidic Microdomains via an Endocytosis-dependent Pathway. Neuron 79, 447–460. 10.1016/j.neuron.2013.05.035 23931995 PMC3741682

[B14] Das U. Wang L. Ganguly A. Saikia J. M. Wagner S. L. Koo E. H. (2016). Visualizing APP and BACE-1 Approximation in Neurons Yields Insight into the Amyloidogenic Pathway. Nat. Neurosci. 19, 55–64. 10.1038/nn.4188 26642089 PMC4782935

[B15] Davis-Salinas J. Saporito-Irwin S. M. Cotman C. W. VAN Nostrand W. E. (1995). Amyloid Beta-Protein Induces its Own Production in Cultured Degenerating Cerebrovascular Smooth Muscle Cells. J. Neurochem. 65, 931–934. 10.1046/j.1471-4159.1995.65020931.x 7616257

[B16] Faghihi M. A. Modarresi F. Khalil A. M. Wood D. E. Sahagan B. G. Morgan T. E. (2008). Expression of a Noncoding RNA Is Elevated in Alzheimer's Disease and Drives Rapid Feed-Forward Regulation of β-secretase. Nat. Med. 14, 723–730. 10.1038/nm1784 18587408 PMC2826895

[B17] Fogel H. Frere S. Segev O. Bharill S. Shapira I. Gazit N. (2014). APP Homodimers Transduce an Amyloid-β-Mediated Increase in Release Probability at Excitatory Synapses. Cell Rep. 7, 1560–1576. 10.1016/j.celrep.2014.04.024 24835997

[B18] Haass C. Kaether C. Thinakaran G. Sisodia S. (2012). Trafficking and Proteolytic Processing of APP. Cold Spring Harbor Perspect. Med. 2, a006270. 10.1101/cshperspect.a006270 PMC3331683 22553493

[B19] Haass C. Lemere C. A. Capell A. Citron M. Seubert P. Schenk D. (1995). The Swedish Mutation Causes Early-Onset Alzheimer's Disease by β-secretase Cleavage within the Secretory Pathway. Nat. Med. 1, 1291–1296. 10.1038/nm1295-1291 7489411

[B20] Harris J. A. Devidze N. Verret L. Ho K. Halabisky B. Thwin M. T. (2010). Transsynaptic Progression of Amyloid-β-Induced Neuronal Dysfunction within the Entorhinal-Hippocampal Network. Neuron 68, 428–441. 10.1016/j.neuron.2010.10.020 21040845 PMC3050043

[B21] Hauser A. S. Attwood M. M. Rask-Andersen M. Schiöth H. B. Gloriam D. E. (2017). Trends in GPCR Drug Discovery: New Agents, Targets and Indications. Nat. Rev. Drug Discov. 16, 829–842. 10.1038/nrd.2017.178 29075003 PMC6882681

[B22] Heredia L. Lin R. Vigo F. S. Kedikian G. Busciglio J. Lorenzo A. (2004). Deposition of Amyloid Fibrils Promotes Cell-Surface Accumulation of Amyloid β Precursor Protein. Neurobiol. Dis. 16, 617–629. 10.1016/j.nbd.2004.04.015 15262274

[B23] Jiang S. Li Y. Zhang X. Bu G. Xu H. Zhang Y.-w. (2014). Trafficking Regulation of Proteins in Alzheimer's Disease. Mol. Neurodegeneration 9, 6. 10.1186/1750-1326-9-6 PMC3891995 24410826

[B24] Kamenetz F. Tomita T. Hsieh H. Seabrook G. Borchelt D. Iwatsubo T. (2003). APP Processing and Synaptic Function. Neuron 37, 925–937. 10.1016/s0896-6273(03)00124-7 12670422

[B25] Kedikian G. Heredia F. Salvador V. R. Raimunda D. Isoardi N. Heredia L. (2010). Secreted Amyloid Precursor Protein and Holo-APP Bind Amyloid Beta through Distinct Domains Eliciting Different Toxic Responses on Hippocampal Neurons. J. Neurosci. Res. 88, 1795–1803. 10.1002/jnr.22347 20155808

[B26] Kerppola T. K. (2006). Design and Implementation of Bimolecular Fluorescence Complementation (BiFC) Assays for the Visualization of Protein Interactions in Living Cells. Nat. Protoc. 1, 1278–1286. 10.1038/nprot.2006.201 17406412 PMC2518326

[B27] Kinoshita A. Fukumoto H. Shah T. Whelan C. M. Irizarry M. C. Hyman B. T. (2003). Demonstration by FRET of BACE Interaction with the Amyloid Precursor Protein at the Cell Surface and in Early Endosomes. J. Cell Sci 116, 3339–3346. 10.1242/jcs.00643 12829747

[B28] Kirouac L. Rajic A. J. Cribbs D. H. Padmanabhan J. (2017). Activation of Ras-ERK Signaling and GSK-3 by Amyloid Precursor Protein and Amyloid Beta Facilitates Neurodegeneration in Alzheimer's Disease. eNeuro 4. 10.1523/ENEURO.0149-16.2017 PMC5367084 28374012

[B29] Koch W. J. Hawes B. E. Allen L. F. Lefkowitz R. J. (1994). Direct Evidence that Gi-Coupled Receptor Stimulation of Mitogen-Activated Protein Kinase Is Mediated by G Beta Gamma Activation of P21ras. Proc. Natl. Acad. Sci. 91, 12706–12710. 10.1073/pnas.91.26.12706 7809106 PMC45508

[B30] Kurt M. A. Davies D. C. Kidd M. (1999). β-Amyloid Immunoreactivity in Astrocytes in Alzheimer's Disease Brain Biopsies: An Electron Microscope Study. Exp. Neurol. 158, 221–228. 10.1006/exnr.1999.7096 10448435

[B31] Lambert M. P. Velasco P. T. Chang L. Viola K. L. Fernandez S. Lacor P. N. (2007). Monoclonal Antibodies that Target Pathological Assemblies of Aβ. J. Neurochem. 100, 23–35. 10.1111/j.1471-4159.2006.04157.x 17116235

[B32] Langer F. Eisele Y. S. Fritschi S. K. Staufenbiel M. Walker L. C. Jucker M. (2011). Soluble A Seeds Are Potent Inducers of Cerebral -Amyloid Deposition. J. Neurosci. 31, 14488–14495. 10.1523/jneurosci.3088-11.2011 21994365 PMC3229270

[B33] Lazarov O. Lee M. Peterson D. A. Sisodia S. S. (2002). Evidence that Synaptically Released β-Amyloid Accumulates as Extracellular Deposits in the Hippocampus of Transgenic Mice. J. Neurosci. 22, 9785–9793. 10.1523/jneurosci.22-22-09785.2002 12427834 PMC6757836

[B34] Lehmann D. M. Seneviratne A. M. P. B. Smrcka A. V. (2008). Small Molecule Disruption of G Protein βγ Subunit Signaling Inhibits Neutrophil Chemotaxis and Inflammation. Mol. Pharmacol. 73, 410–418. 10.1124/mol.107.041780 18006643 PMC2742223

[B35] Libeu C. P. Poksay K. S. John V. Bredesen D. E. (2011). Structural and Functional Alterations in Amyloid-β Precursor Protein Induced by Amyloid-β Peptides. Jad 25, 547–566. 10.3233/jad-2011-101938 21471643 PMC4001850

[B36] Lorenzo A. Yuan M. Zhang Z. Paganetti P. A. Sturchler-Pierrat C. Staufenbiel M. (2000). Amyloid β Interacts with the Amyloid Precursor Protein: a Potential Toxic Mechanism in Alzheimer's Disease. Nat. Neurosci. 3, 460–464. 10.1038/74833 10769385

[B37] Mamada N. Tanokashira D. Hosaka A. Kametani F. Tamaoka A. Araki W. (2015). Amyloid β-protein Oligomers Upregulate the β-secretase, BACE1, through a post-translational Mechanism Involving its Altered Subcellular Distribution in Neurons. Mol. Brain 8, 73. 10.1186/s13041-015-0163-5 26552445 PMC4638102

[B38] Marinissen M. J. Gutkind J. S. (2001). G-protein-coupled Receptors and Signaling Networks: Emerging Paradigms. Trends Pharmacol. Sci. 22, 368–376. 10.1016/s0165-6147(00)01678-3 11431032

[B39] Marsden I. T. Minamide L. S. Bamburg J. R. (2011). Amyloid-β-Induced Amyloid-β Secretion: A Possible Feed-Forward Mechanism in Alzheimer's Disease. Jad 24, 681–691. 10.3233/jad-2011-101899 21297255 PMC4447202

[B40] Nakamura N. Rabouille C. Watson R. Nilsson T. Hui N. Slusarewicz P. (1995). Characterization of a Cis-Golgi Matrix Protein, GM130. J. Cell Biol 131, 1715–1726. 10.1083/jcb.131.6.1715 8557739 PMC2120691

[B41] Nishimoto I. Okamoto T. Matsuura Y. Takahashi S. Okamoto T. Murayama Y. (1993). Alzheimer Amyloid Protein Precursor Complexes with Brain GTP-Binding Protein Go. Nature 362, 75–79. 10.1038/362075a0 8446172

[B42] Oddo S. Caccamo A. Shepherd J. D. Murphy M. P. Golde T. E. Kayed R. (2003). Triple-Transgenic Model of Alzheimer's Disease with Plaques and Tangles. Neuron 39, 409–421. 10.1016/s0896-6273(03)00434-3 12895417

[B43] Puzzo D. Piacentini R. Fá M. Gulisano W. Li Puma D. D. Staniszewski A. (2017). LTP and Memory Impairment Caused by Extracellular Aβ and Tau Oligomers Is APP-dependent. Elife 6, e26991. 10.7554/eLife.26991 28696204 PMC5529106

[B44] Rajendran L. Honsho M. Zahn T. R. Keller P. Geiger K. D. Verkade P. (2006). Alzheimer's Disease β-amyloid Peptides Are Released in Association with Exosomes. Proc. Natl. Acad. Sci. U.S.A. 103, 11172–11177. 10.1073/pnas.0603838103 16837572 PMC1544060

[B45] Sadleir K. R. Vassar R. (2012). Cdk5 Protein Inhibition and Aβ42 Increase BACE1 Protein Level in Primary Neurons by a Post-transcriptional Mechanism. J. Biol. Chem. 287, 7224–7235. 10.1074/jbc.m111.333914 22223639 PMC3293556

[B46] Sannerud R. Declerck I. Peric A. Raemaekers T. Menendez G. Zhou L. (2011). ADP Ribosylation Factor 6 (ARF6) Controls Amyloid Precursor Protein (APP) Processing by Mediating the Endosomal Sorting of BACE1. Proc. Natl. Acad. Sci. U S A. 108, E559–E568. 10.1073/pnas.1100745108 21825135 PMC3161548

[B47] Selkoe D. J. Hardy J. (2016). The Amyloid Hypothesis of Alzheimer's Disease at 25 Years. EMBO Mol. Med. 8, 595–608. 10.15252/emmm.201606210 27025652 PMC4888851

[B48] Seneviratne A. M. P. B. Burroughs M. Giralt E. Smrcka A. V. (2011). Direct-reversible Binding of Small Molecules to G Protein βγ Subunits. Biochim. Biophys. Acta (Bba) - Proteins Proteomics 1814, 1210–1218. 10.1016/j.bbapap.2011.05.010 21621014 PMC3140432

[B49] Shaked G. M. Kummer M. P. Lu D. C. Galvan V. Bredesen D. E. Koo E. H. (2006). Aβ Induces Cell Death by Direct Interaction with its Cognate Extracellular Domain on APP (APP 597-624). FASEB j. 20, 1254–1256. 10.1096/fj.05-5032fje 16636103 PMC1847355

[B50] Sola Vigo F. Kedikian G. Heredia L. Heredia F. Añel A. D. Rosa A. L. (2009). Amyloid-β Precursor Protein Mediates Neuronal Toxicity of Amyloid β through Go Protein Activation. Neurobiol. Aging 30, 1379–1392. 10.1016/j.neurobiolaging.2007.11.017 18187234

[B51] Stine W. B. JR. Dahlgren K. N. Krafft G. A. Ladu M. J. (2003). *In Vitro* Characterization of Conditions for Amyloid-β Peptide Oligomerization and Fibrillogenesis. J. Biol. Chem. 278, 11612–11622. 10.1074/jbc.m210207200 12499373

[B52] Takahashi R. H. Milner T. A. Li F. Nam E. E. Edgar M. A. Yamaguchi H. (2002). Intraneuronal Alzheimer Aβ42 Accumulates in Multivesicular Bodies and Is Associated with Synaptic Pathology. Am. J. Pathol. 161, 1869–1879. 10.1016/s0002-9440(10)64463-x 12414533 PMC1850783

[B53] Takahashi R. H. NAGAO T. GOURAS G. K. (2017). Plaque formation and the intraneuronal accumulation of β-amyloid in Alzheimer's disease. Pathol Int 67, 185–193. 10.1111/pin.12520 28261941

[B54] Thal D. R. Härtig W. Schober R. (1999). Diffuse Plaques in the Molecular Layer Show Intracellular A Beta(8-17)-Immunoreactive Deposits in Subpial Astrocytes. Clin. Neuropathol. 18, 226–231. 10505431

[B55] Thal D. R. Rüb U. Orantes M. Braak H. (2002). Phases of Aβ-Deposition in the Human Brain and its Relevance for the Development of AD. Neurology 58, 1791–1800. 10.1212/wnl.58.12.1791 12084879

[B56] Thinakaran G. Koo E. H. (2008). Amyloid Precursor Protein Trafficking, Processing, and Function. J. Biol. Chem. 283, 29615–29619. 10.1074/jbc.r800019200 18650430 PMC2573065

[B57] Thinakaran G. Teplow D. B. Siman R. Greenberg B. Sisodia S. S. (1996). Metabolism of the "Swedish" Amyloid Precursor Protein Variant in Neuro2a (N2a) Cells. J. Biol. Chem. 271, 9390–9397. 10.1074/jbc.271.16.9390 8621605

[B58] Ullrich O. Reinsch S. Urbé S. Zerial M. Parton R. G. (1996). Rab11 Regulates Recycling through the Pericentriolar Recycling Endosome. J. Cell Biol 135, 913–924. 10.1083/jcb.135.4.913 8922376 PMC2133374

[B59] Van Nostrand W. E. Melchor J. P. Keane D. M. Saporito-Irwin S. M. Romanov G. Davis J. (2002). Localization of a Fibrillar Amyloid β-Protein Binding Domain on its Precursor. J. Biol. Chem. 277, 36392–36398. 10.1074/jbc.m204676200 12107175

[B60] Vassar R. Bennett B. D. Babu-Khan S. Kahn S. Mendiaz E. A. Denis P. (1999). β-Secretase Cleavage of Alzheimer's Amyloid Precursor Protein by the Transmembrane Aspartic Protease BACE. Science 286, 735–741. 10.1126/science.286.5440.735 10531052

[B61] Walker L. C. Jucker M. (2015). Neurodegenerative Diseases: Expanding the Prion Concept. Annu. Rev. Neurosci. 38, 87–103. 10.1146/annurev-neuro-071714-033828 25840008 PMC4803040

[B62] Wang Z. Jackson R. J. Hong W. Taylor W. M. Corbett G. T. Moreno A. (2017). Human Brain-Derived Aβ Oligomers Bind to Synapses and Disrupt Synaptic Activity in a Manner that Requires APP. J. Neurosci. 37, 11947–11966. 10.1523/jneurosci.2009-17.2017 29101243 PMC5719975

[B63] Yang A. J. Chandswangbhuvana D. Shu T. Henschen A. Glabe C. G. (1999). Intracellular Accumulation of Insoluble, Newly Synthesized Aβn-42 in Amyloid Precursor Protein-Transfected Cells that Have Been Treated with Aβ1-42. J. Biol. Chem. 274, 20650–20656. 10.1074/jbc.274.29.20650 10400697

[B64] Zeng H. Guo M. Martins-Taylor K. Wang X. Zhang Z. Park J. W. (2010). Specification of Region-specific Neurons Including Forebrain Glutamatergic Neurons from Human Induced Pluripotent Stem Cells. PLoS One 5, e11853. 10.1371/journal.pone.0011853 20686615 PMC2912324

[B65] Zhao J. O'Connor T. Vassar R. (2011). The Contribution of Activated Astrocytes to Aβ Production: Implications for Alzheimer's Disease Pathogenesis. J. Neuroinflammation 8, 150. 10.1186/1742-2094-8-150 22047170 PMC3216000

