# Supplementary figures and images for: Aβ Assemblies Promote Amyloidogenic Processing of APP and Intracellular Accumulation of Aβ42 Through Go/Gβγ Signaling
Source: Front Cell Dev Biol. 2022 Apr 4;10:852738. doi: 10.3389/fcell.2022.852738 (PMC9013780; doi:10.3389/fcell.2022.852738)

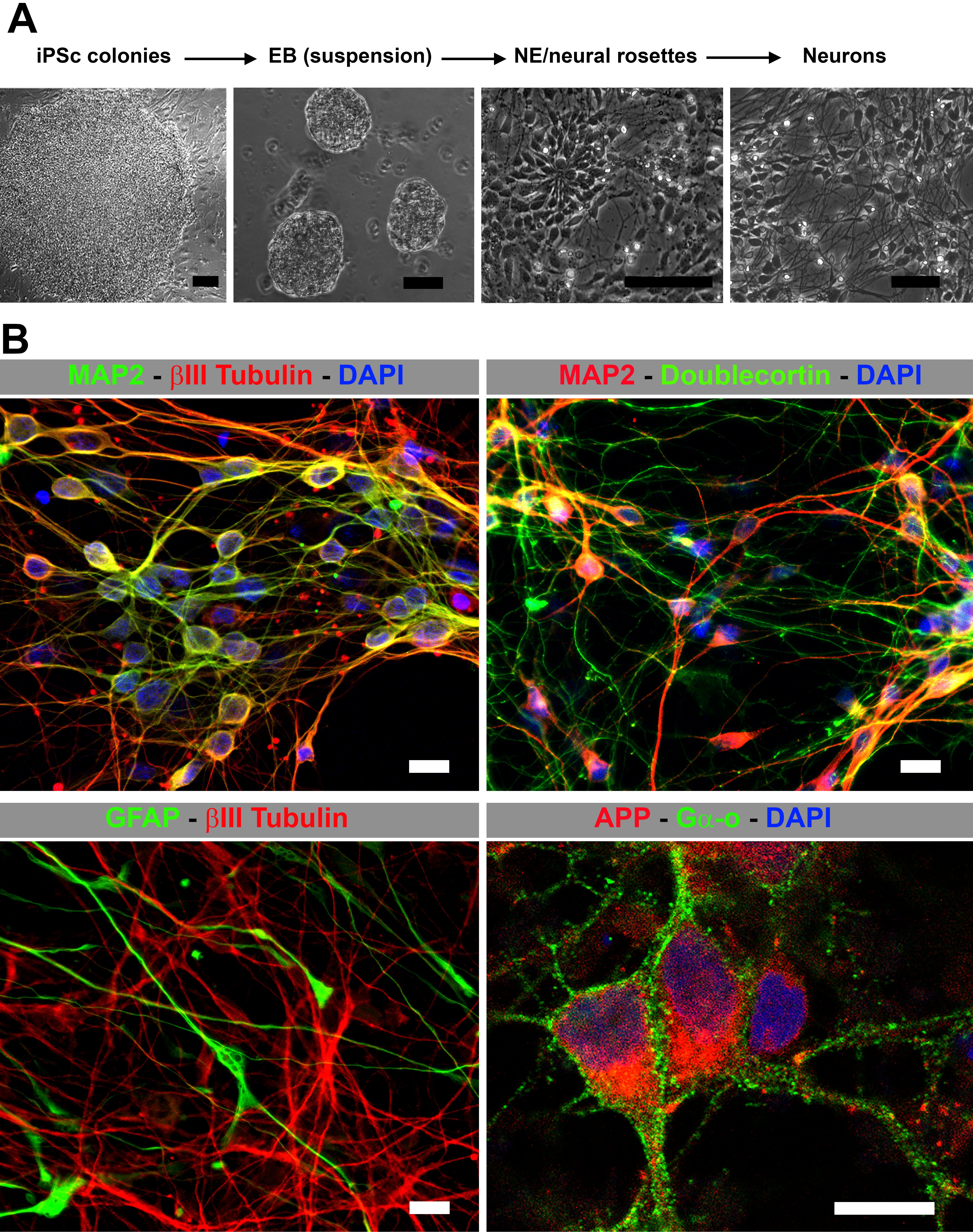

Supplement: Supplementary file 1 [file Image3.JPEG]

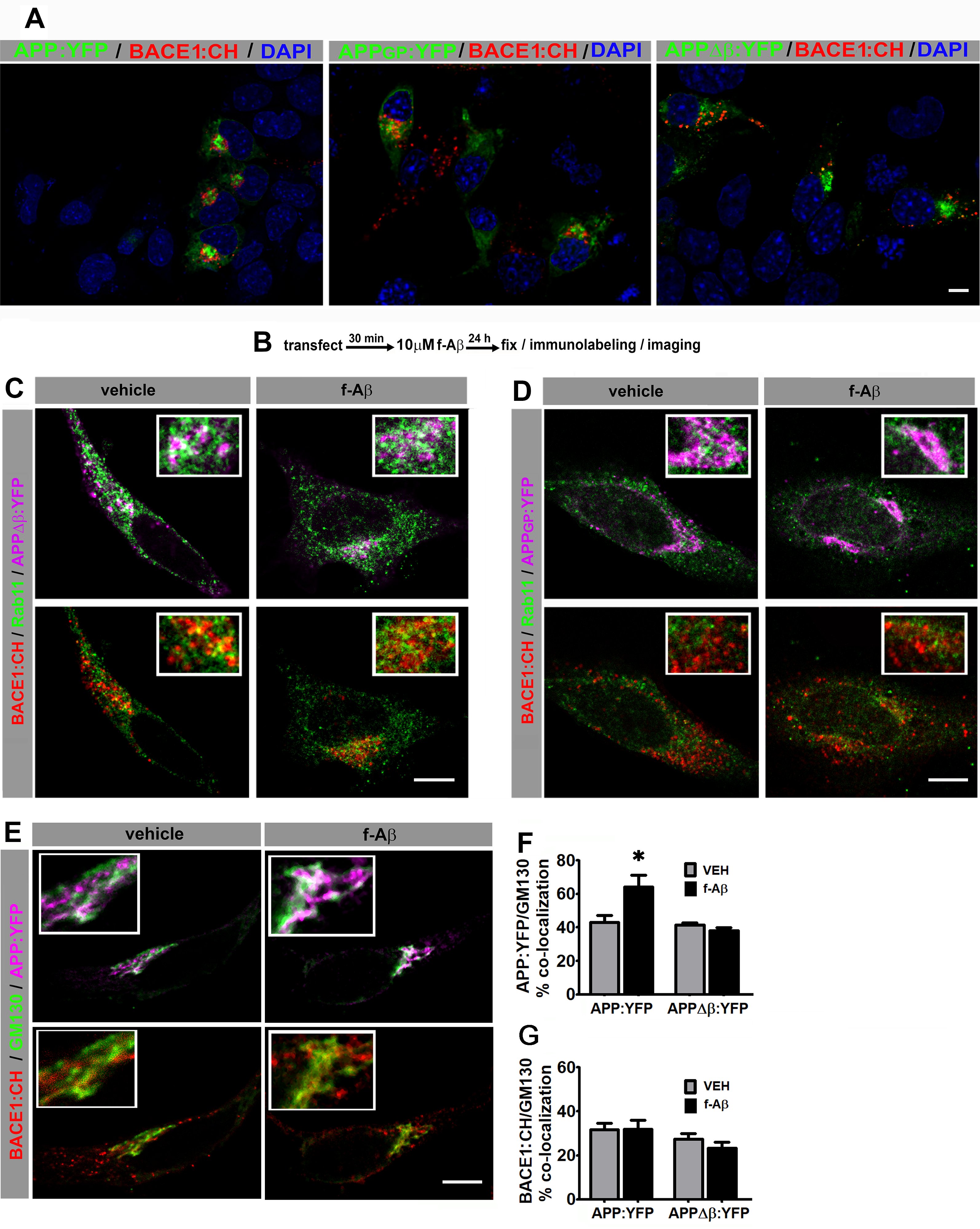

Supplement: Supplementary file 2 [file Image1.JPEG]

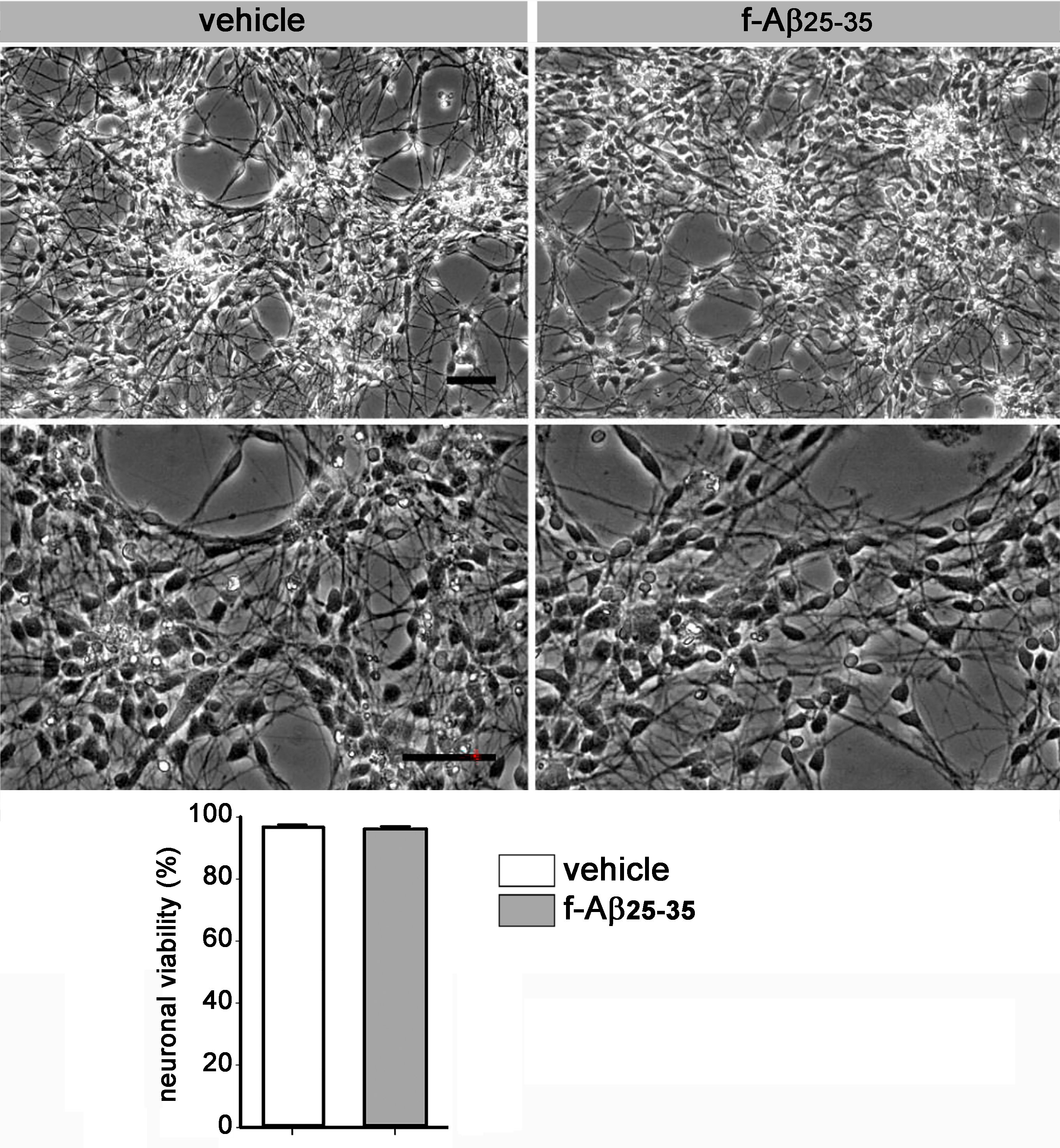

Supplement: Supplementary file 3 [file Image4.JPEG]

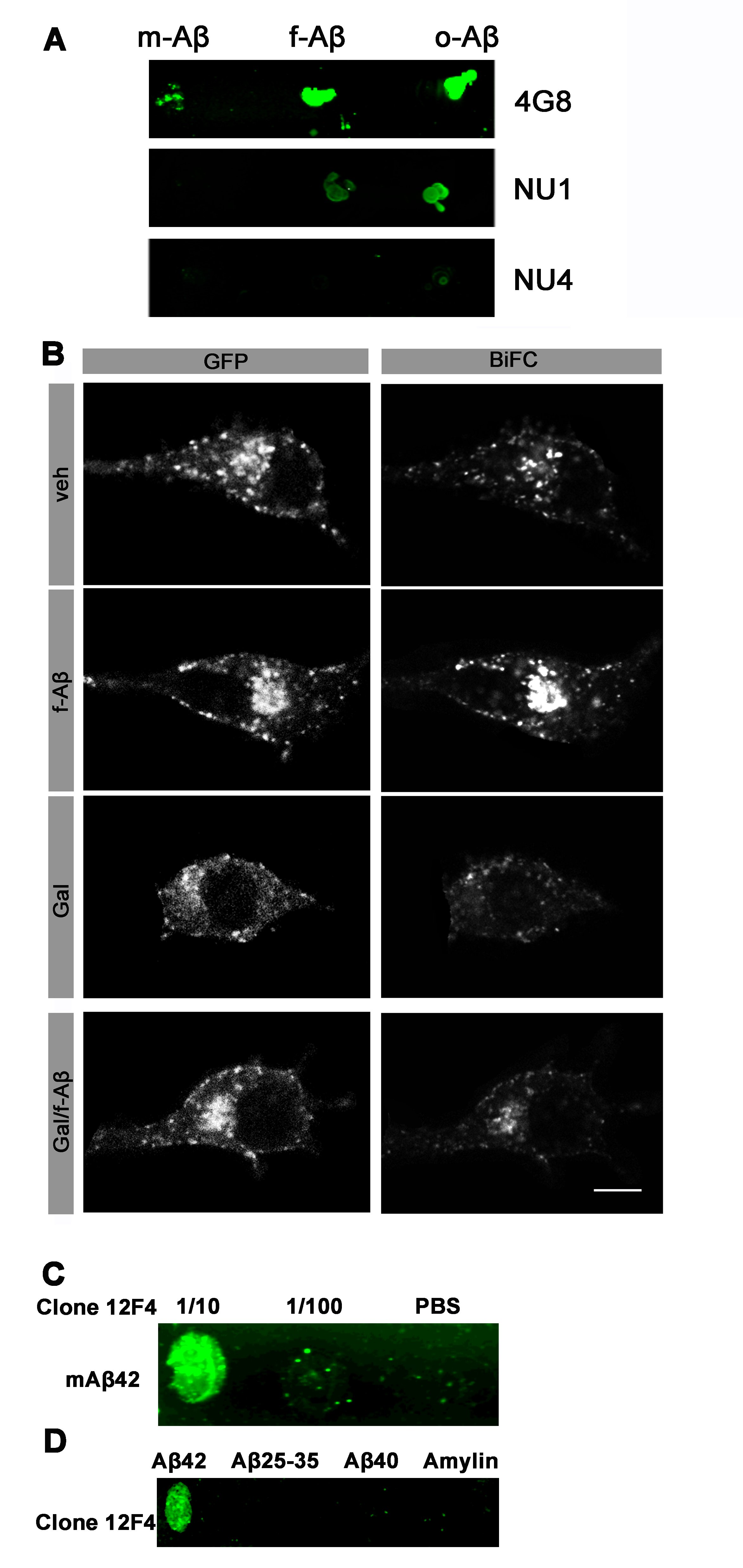

Supplement: Supplementary file 4 [file Image2.JPEG]
